# Probabilistic projection of subnational total fertility rates

**DOI:** 10.4054/DemRes.2018.38.60

**Published:** 2018-06-08

**Authors:** Hana Ševčíková, Adrian E. Raftery, Patrick Gerland

**Affiliations:** 1 Center for Statistics and the Social Sciences, University of Washington, Seattle, USA.; 2 Departments of Statistics and Sociology, University of Washington, Seattle, USA.; 3 United Nations Population Division, United Nations, New York, USA.

## Abstract

**BACKGROUND:**

We consider the problem of probabilistic projection of the total fertility rate (TFR) for subnational regions.

**OBJECTIVE:**

We seek a method that is consistent with the UN’s recently adopted Bayesian method for probabilistic TFR projections for all countries and works well for all countries.

**METHODS:**

We assess various possible methods using subnational TFR data for 47 countries.

**RESULTS:**

We find that the method that performs best in terms of out-of-sample predictive performance and also in terms of reproducing the within-country correlation in TFR is a method that scales each national trajectory from the national predictive posterior distribution by a region-specific scale factor that is allowed to vary slowly over time.

**CONCLUSIONS:**

Probabilistic projections of TFR for subnational units are best produced by scaling the national projection by a slowly time-varying region-specific scale factor. This supports the hypothesis of Watkins (1990, 1991) that within-country TFR converges over time in response to country-specific factors, and thus extends the Watkins hypothesis to the last 50 years and to a much wider range of countries around the world.

**CONTRIBUTION:**

We have developed a new method for probabilistic projection of subnational TFR that works well and outperforms other methods. This also sheds light on the extent to which within-country TFR converges over time.

## 1. Introduction

The United Nations Population Division issued official probabilistic population projections for all countries for the first time in 2015 (United Nations 2015), using the methodology described by Raftery et al. (2012). One of the key components of the projection methodology is a Bayesian hierarchical model for the total fertility rate (TFR) in all countries (Alkema et al. 2011; Raftery, Alkema, and Gerland 2014; Fosdick and Raftery 2014).

Population projections for subnational administrative units, such as provinces, states, counties, regions, or départements (hereafter all referred to simply as regions), are of great interest to national and local governments for planning, policy, and decision-making (Rayer, Smith, and Tayman 2009). Typically these are used by policy and decision-makers at the national or subnational level.

A common current practice is to generate subnational projections deterministically by scaling national projections (US Census Bureau 2016). Specifically, the US Census Bureau provides a workbook for users to generate subnational TFR projections for up to 32 regions. The method requires the user to enter an ultimate TFR level (lower asymptote), to which the regional TFR converges, and a deterministic projection of the national TFR. The subnational TFR is then projected in such a way that it approaches the target TFR at the same rate as the national TFR approaches this target. The methods used by several other national agencies were reviewed by Rees et al. (2015), including methods used in Wales (Statistics for Wales 2017), Northern Ireland (NISRA 2014), and Canada (Statistics Canada 2014). These methods do not yield probabilistic projections.

In this paper we try to address one aspect of the problem, namely probabilistic sub-national projections of TFR. Methods for probabilistic subnational projections have been developed for individual countries or parts of countries (Smith and Sincich 1988; Tayman, Schafer, and Carter 1998; Rees and Turton 1998; Gullickson and Moen 2001; Gullickson 2001; Lee, Miller, and Edwards 2003; Smith and Tayman 2004; Wilson and Bell 2007; Rayer, Smith, and Tayman 2009; Raymer, Abel, and Rogers 2012; Wilson 2013); for a review see Tayman (2011). Our ultimate goal is to extend the UN method for probabilistic projections for all countries to a method for subnational probabilistic projections that is consistent across countries and that works well for all regions of all countries. In practice, we anticipate that this method would be used mostly by national or subnational-level policy-makers for their own country or region. However, we have developed our method using data from multiple and diverse countries in the hope that the method would be useful for decision-makers in a wide range of countries with different circumstances.

We contrast two broad approaches to subnational probabilistic projection of TFR. One approach is a direct extension of the UN method (Alkema et al. 2011) to subnational data, effectively treating the country in the same way the UN model treats the world, and treating the regions in the same way the UN model treats the countries. Borges (2015) proposed an approach along these lines for the provinces of Brazil.

The other approach is motivated by the observation of Watkins (1990, 1991) that within-country variation in TFR in Europe decreased over the period of the fertility transition there, between 1870 and 1960. This observation has been confirmed for a more recent period for the German-speaking countries (Basten, Huinink, and Klüsener 2012), to some extent for India (Arokiasmy and Goli 2012; Wilson et al. 2012), while the evidence is more equivocal for the United States (O’Connell 1981). Watkins posits that this was due to increased integration of national markets, expansion of the role of the state, and nation-building in the form of linguistic standardization over this period. Calhoun (1993) argues that, of these three mechanisms, only linguistic standardization clearly supports her argument.

However, some support for the importance of the role of the nation state for fertility is provided by the fact that nation states have specific and different policies aimed at affecting fertility rates (Tomlinson 1985; Chamie 1994), and some of these policies have been shown to be effective (Kalwij 2010; Luci-Greulich and Thévenon 2013). Gauthier (2007) argues on the other hand that family policies have little impact. Note that Klüsener, Perelli-Harris, and Gassen (2013) investigated subnational convergence of non-marital fertility in Europe in recent decades and found that within-country variation increased. Similarly, de Beer and Deerenberg (2007) used a regression model to project differences in the level of fertility between Dutch municipalities and concluded that fertility is not likely to converge. These results are in contrast with the trends noted by other authors.

One question is then whether the direct extension of the UN method for countries to the subnational context adequately accounts for this tendency of TFR to converge within countries over time. Note that this extension of the UN method does predict within-country convergence of fertility rates over time during the fertility transition; the question is whether it adequately accounts for this convergence.

To investigate this question, we consider a different general approach, which starts from the national probabilistic projections produced by the UN method and then scales them for each region by a scaling factor that varies stochastically, but stays relatively constant. This induces more within-country correlation than the direct extension of the UN method. It could be viewed as a probabilistic extension of the method currently used by the US Census Bureau. It is also related to the method of Wilson (2013), but with some significant differences.

We apply these methods to subnational data on total fertility for 47 countries over the period 1950–2010. We compare our two approaches and several variants in terms of out-of-sample predictive performance. The results shed some light on the Watkins hypothesis of increasing within-country correlation, and they provide some guidance on how to carry out subnational probabilistic TFR projection.

Note that there is substantial literature on convergence of fertility rates in different countries to one another, with different conclusions argued (Wilson 2001, 2004; Reher 2004, 2007; Dorius 2008; Wilson 2011). Our work here has implications for within-country fertility convergence, but it is agnostic about fertility convergence between countries and so does not have implications for global fertility convergence, for example.

The paper is organized as follows. We first describe the data used in this study and review the model for national probabilistic projections. We then introduce our proposed methodology for subnational probabilistic projections and present the results. The paper concludes with a discussion.

## 2. Data

**Figure 1:**
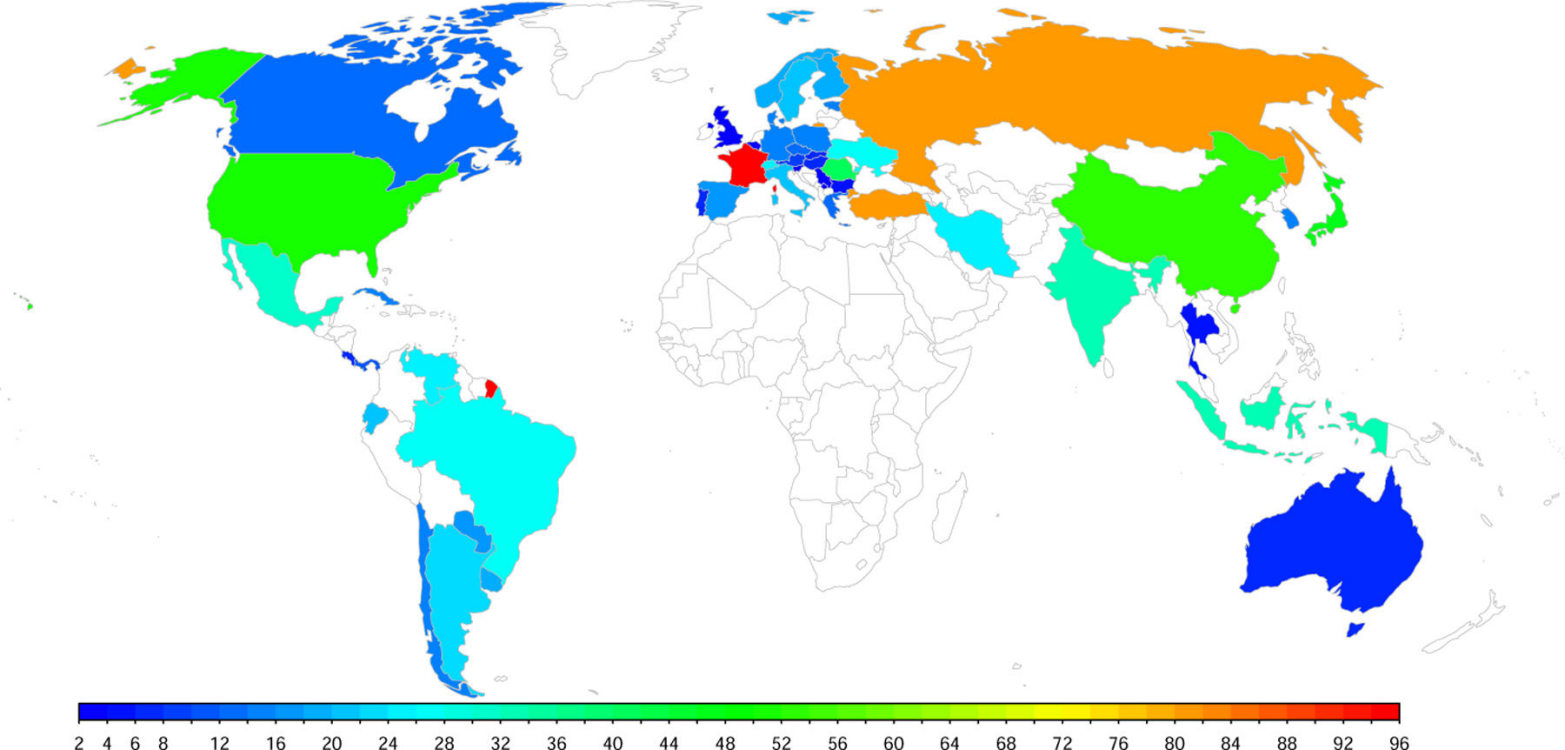
Map of 47 countries with subnational TFR data *Note:* The color scale shows the number of regions for each country, which ranges from 2 to 96.

We use available subnational data on the TFR for 47 countries (13 in the Americas, 9 in the Asia-Pacific region, and 25 in Europe), corresponding to 1,092 regions for the period 1950–2010. Each country analyzed had a population over one million and a national average TFR below 2.5 in 2010–2015. The geographical level selected for each country was the one with available data for the longest comparable time series. The dataset covers 4.9 billion people, or about two-thirds of the world’s population. Figure 1 shows the numbers of regions for each country, which range from 2 for Slovenia to 96 for France. The data includes countries from all the inhabited continents except Africa. The data sources are shown in Appendix Table A-3. Note that, while estimates are available for all countries at the national level to 2015 (United Nations 2017), the data we are using for all regions of the countries we analyze has been collated only until 2010. The dataset is available at https://bayespop.csss.washington.edu/download/#subnatTFR.

For statistical purposes, Eurostat has developed a Nomenclature of Territorial Units for Statistics for the European Union (NUTS, http://ec.europa.eu/eurostat/web/nuts/overview). No equivalent statistical nomenclature exists at the international level for other regions, but we provide a NUTS-equivalent assessment for regions outside Europe to assist the comparison between countries.

The subnational data used in this analysis has been compiled by the authors and are based on national data sources described in Table A-3 for each country. The reliability of this data varies between countries, but for a majority of the countries the fertility estimates are based on birth registration data. For Asian and Latin American countries which lack nationally representative vital registration, these fertility estimates are based on surveys and censuses. For all European countries for which Eurostat series are available, this data has been used, unless longer time series were available directly from national data sources.

The selection of countries used for this analysis is based on a combination of factors: (1) availability of fertility rates at subnational level for a meaningful length of time, (2) reasonably stable geographical divisions over time allowing meaningful comparisons, (3) sufficient population size to allow subnational disaggregation, (4) a range of countries covering different regions, to the extent possible, and (5) having completed most or all of the Phase II of their fertility transition at the national level.

In terms of geographic coverage, the list of countries included in this analysis is reasonably comprehensive based on criteria 1–3 for all countries with nationally representative vital registration publishing fertility rates by subnational divisions. Several additional European countries could not be included in this analysis due to factors 1–3 that created substantial breaks in the time series due to major administrative changes or availability only for the most recent decade (e.g., Albania, Belarus, Bosnia and Herzegovina, Croatia, Ireland, Latvia, Luxembourg, Malta, Montenegro, Netherlands, TFYR Macedonia).

**Figure 2:**
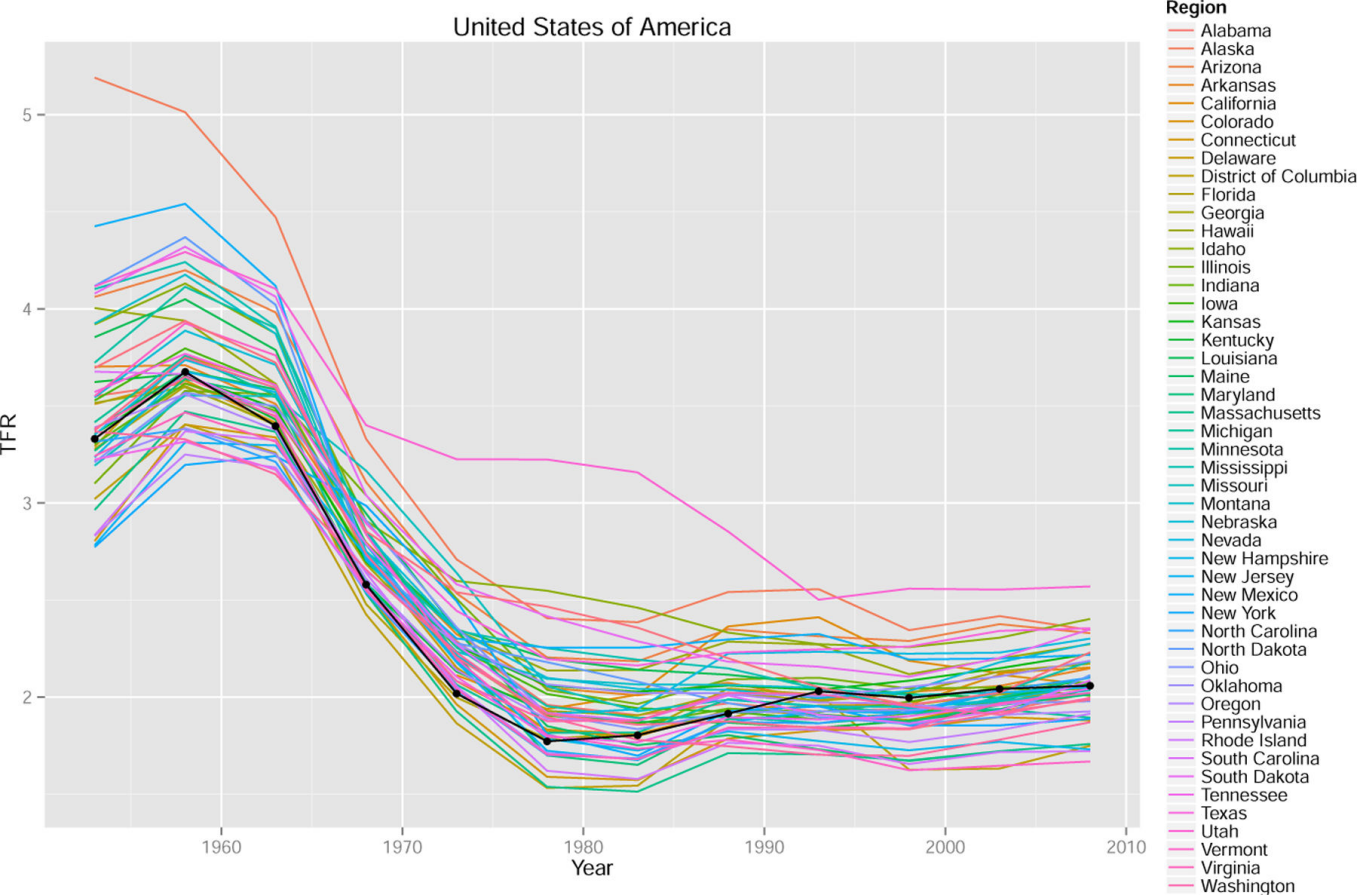

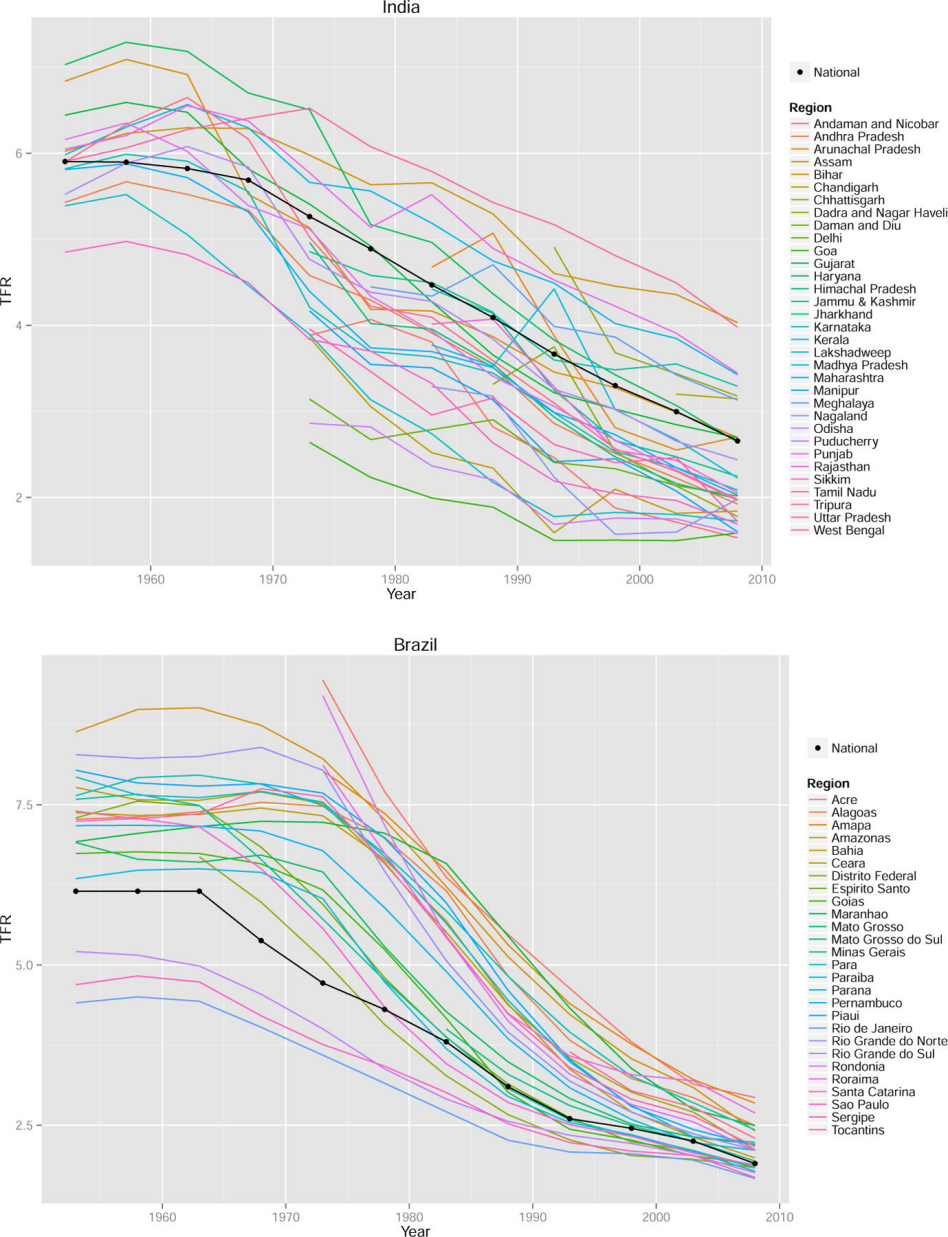

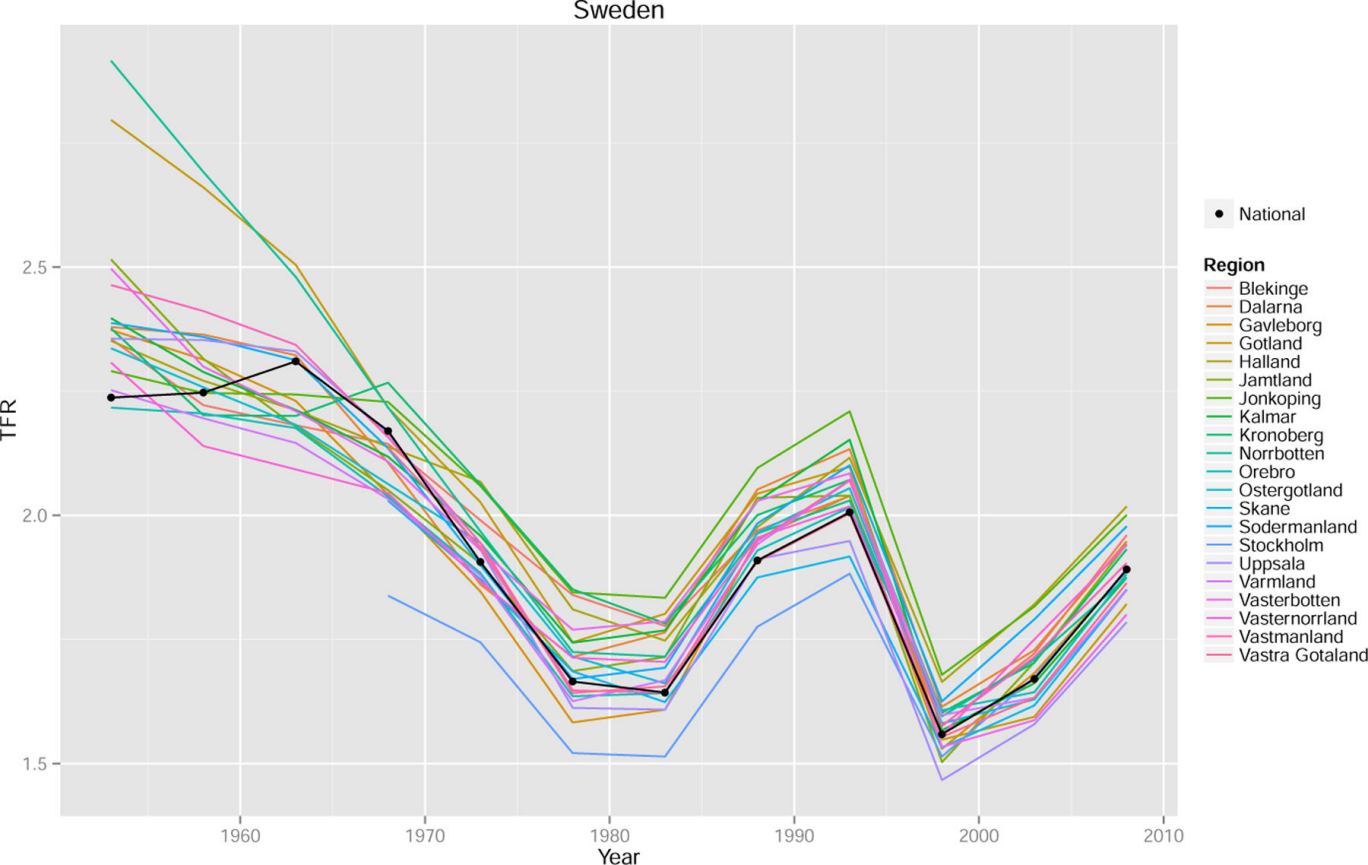
Observed data for regions of the United States, India, Brazil, and Sweden Note: The national TFR is shown by the black curve.

Figure 2 shows an example of the data for four countries (United States, India, Brazil, and Sweden). It illustrates that the data varies with respect to the correlation between regions. It also shows that the data started later than 1950 for some regions. In the figure, the national TFR from United Nations (2013) is shown as a black curve.

## 3. Review of the national Bayesian hierarchical model

Our starting point for developing a methodology for subnational projections is the probabilistic model for projecting national TFR proposed by Alkema et al. (2011), which has now been adopted by the UN for its official projections. We start by summarizing the main ideas of this Bayesian hierarchical model (BHM). More detail can be found in Alkema et al. (2011) and Raftery, Alkema, and Gerland (2014).

**Figure 3:**
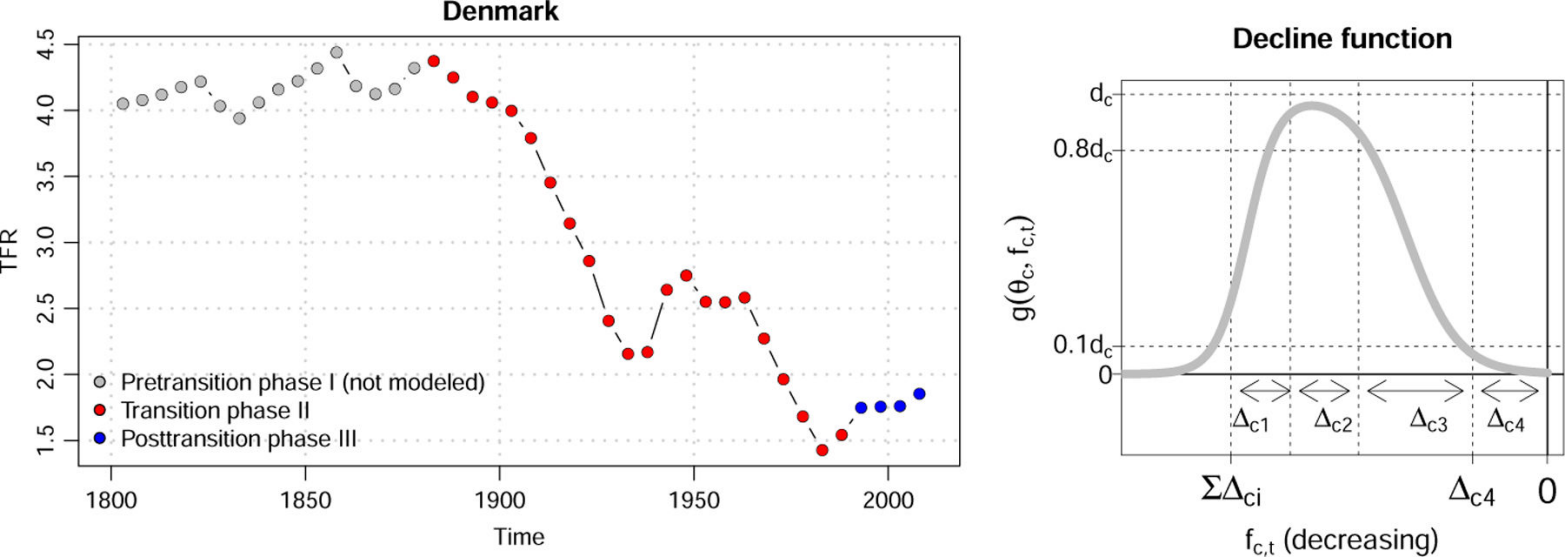
Three phases of the typical TFR evolution for the example of Denmark (left); cartoon of a double logistic decline curve (right) *Note*: The right panel shows a double logistic decline curve for country *c* with its parameters defining the shape. *f*_*c,t*_ on the *x* axis denotes the TFR, while *g*(*θ*_*c*_, *f*_*c,t*_) on the *y* axis denotes the first order difference in TFR.

The model is based on standard fertility transition theory (Hirschman 1994), and it is compatible with almost all versions of this in the literature. It distinguishes three phases in the evolution of a country’s fertility over time, depicted in the left panel of Figure 3 for the example of Denmark. Phase I (grey dots) precedes the beginning of the fertility transition and is characterized by high fertility that is stable or increasing. This phase is not modeled as all or nearly all countries have completed this phase. During Phase II, or the transition phase (red dots in the figure), fertility declines from high levels to below the replacement level of 2.1 children per woman. Phase III is the post-fertility transition period (blue dots), during which fertility fluctuates at low levels, possibly recovering towards the replacement level.

To model the fertility declines in each five-year period during Phase II, a double logistic decline function is used. An example of this function is shown in the right panel of Figure 3. The function is parametrized by a set of country-specific parameters that define the shape of the country’s decline curve. Those parameters are drawn from a world distribution. The resulting BHM is estimated using Markov chain Monte Carlo (MCMC).

Phase III is modeled using a Bayesian hierarchical first-order autoregressive, or AR(1), process of the form:

$$f_{c,t+1}-\mu_{c}=\rho_{c}\left(f_{c,t}-\mu_{c}\right)+\varepsilon_{c,t},\;\text{with}\;\varepsilon_{c,t}\overset{iid}{\sim }N\left(0,\sigma_{\varepsilon }^{2}\right).$$

It implies that fertility for country *c* has a country-specific long-term mean, *μ*_*c*_, and autoregressive parameter, *ρ*_*c*_, which are assumed to be drawn from a world distribution. The parameters of this world distribution in turn have a joint prior distribution, thus defining a three-level hierarchical model, where the three levels are the observation, the country, and the world. The resulting model is again estimated by MCMC.

The process of estimating Phase II and Phase III parameters results in a set of country-specific decline curves and a set of country-specific AR(1) parameter pairs. Unlike decline curves, which can be estimated for all countries, not all countries have experienced Phase III, in which cases the country-specific long-term means and autoregressive parameters cannot be estimated. In such cases, the ‘world’ means and autoregressive parameters are used. The estimated parameters are then used to generate a set of future TFR trajectories yielding probabilistic TFR projections for all countries of the world.

## 4. Methods for subnational projections

Ideally, we seek a method for generating probabilistic subnational TFR projections that reflects the literature and theory of fertility transitions, is based on the national methodology used by the UN and described above, works well for all countries, is as simple as possible, and yields correlations between regions that are similar to the correlations in the observed data.

We first describe a simple Scale method that provides an initial probabilistic extension of methods used by the US Census Bureau and other national agencies. This simple approach works well from many points of view, but it does not allow for the possibility of crossovers between regions, whereas in fact these do happen. We therefore elaborate this model to allow the scale factor to change stochastically, but slowly over time, yielding the so-called Scale-AR(1) method. Finally we describe a quite different approach, called the one-directional BHM, which directly generalizes the national approach to the subnational context, allowing regions to vary more freely within a country.

### 4.1 Scale method

We start with a simple intuitive scale method where, for each trajectory from the probabilistic projection, the regional TFR is simply a product of the simulated national TFR and a time-independent but region-specific scale factor.

Let *f*_*c,t,i*_ denote the national TFR projection for country *c* at time *t* from trajectory *i*, simulated from its posterior distribution as described above. We model $f_{r_{c},t,i}$, the TFR for region *r*_*c*_ of country *c* at time *t* in the *i*-th trajectory, by

(1) $$f_{r_{c},t,i}=\alpha_{r_{c}}f_{c,t,i},$$

where $\alpha_{r_{c}}$ denotes the regional scaling factor derived from the last observed (present) time period denoted by *P*:

(2) $$\alpha_{r_{c}}=f_{r_{c},t=P}/f_{c,t=P}.$$

Note that $\alpha_{r_{c}}$ is the same for all trajectories. This method yields a set of regional trajectories $f_{r_{c},t,i}$ and thus yields probabilistic projections of the regional TFRs, $f_{r_{c},t}$.

Our numerical experiments, described below, indicated that this simple method performed surprisingly well. However, it also has a serious drawback. Scaling by a constant factor induces a perfect correlation, i.e., it does not allow for the possibility of crossovers between regions over time. However, such crossovers do happen, and the scale method says that they are impossible, which is not fully satisfactory.

### 4.2 Scale-AR(1)

To avoid this drawback, and modify the scale method so as to allow for the possibility of crossovers, we propose a variation of the simple Scale method where we model the regional scale factor using a first-order autoregressive, or AR(1), process:

(3) $$\alpha_{r_{c},t}-1=\phi \left(\alpha_{r_{c},t-1}-1\right)+\varepsilon_{r_{c},t},\;\text{with}\;\varepsilon_{r_{c},t}\overset{iid}{\sim }N\left(0,\sigma_{c}^{2}\right).$$

The regional TFR $f_{r_{c},t,i}$ is then derived as in (1) with the additional lower bound restriction, $f_{r_{c},t,i}>0.5$.

This model implies that the scaling factor will fluctuate around 1 in the long term. Regardless of its initial value, it will converge to a distribution that is centered around 1, and the rate of convergence is determined by the *ϕ* parameter. We use the following settings for the model parameters, estimated from the data for all 47 countries available:

(4) ϕ=0.925,

(5) $$\sigma_{c}^{2}=\min\left\{\sigma^{2},\left(1-\phi^{2}\right){\text{Var}}_{r\in R_{c}}\left(\alpha_{r,t=P}\right)\right\},$$

(6) σ=0.0452,

where *P* again denotes the present time period and *R*_*c*_ denotes the set of regions of country *c*. The minimum restriction in (5) ensures that the variation of *α.*_*,t*_ across regions is not larger than the variation in the last observed time period, in line with the Watkins hypothesis and the long-term observed data.

We experimented with different methods of determining these parameters, including the use of country-specific values. The method we used is based on the asymptotic variance of *α* and yielded the best validation results. Reasonable changes in *ϕ* and *σ* made very little difference to the results. Details of how these parameters were estimated are given in the appendix.

This method is related to the method proposed by Wilson (2013), but there are some significant differences that are discussed in the Discussion section.

### 4.3 One-directional BHM

**Figure 4:**
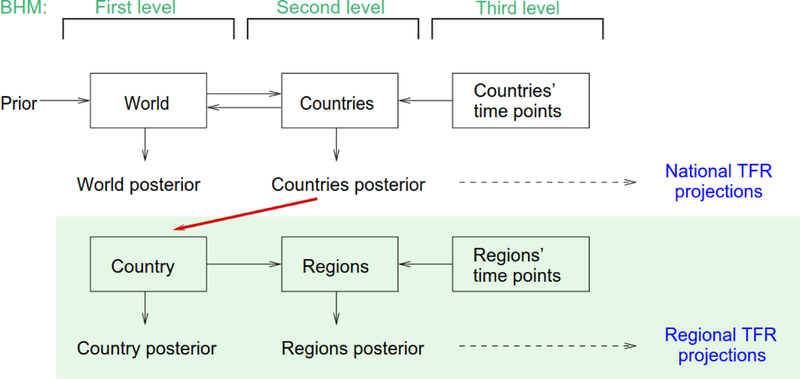
One-directional BHM for the subnational model

Next, we consider an extension of the world three-level BHM, which is depicted in Figure 4. The three levels of this model are the world level, country level, and time point or observation level. In the world version, information from all countries is combined into the world level, which in turn influences the country level, yielding a two-directional BHM. The prior distribution of the hyperparameters is vague for most parameters, but also reflects expert knowledge in some cases. The model yields a posterior distribution of the world parameters, and the country-specific parameters, which is then used to generate the national projections.

Our extension has a similar setup, but moves down by one level of geography and works in one direction only. Thus the top level of our national model is the country, the next level is the region, and the bottom level is the time point. The upper level of our model corresponds to the country level of the world model; that is, we carry over the country-specific posterior from a world simulation and use it as the distribution of the hyperparameters in our national model (red arrow in Figure 4). On the lower level, data from all regions of a country is handled individually. The estimation of the regional parameters is informed by the hyperparameters, but the regional level does not influence the country level of the model. The resulting regional posterior distribution is used to project subnational TFR.

Note that many countries do not have historical data on Phase III because they have not yet reached this stage, and so in these cases the country posterior is the same as the world posterior. As a result, all regions of those countries inherit the ‘world’ Phase III parameters.

### 4.4 Correlation between regions

For aggregating TFR over sets of regions, for example for deriving country’s averages, it is important to capture correlation in model errors between regions of a country, as was done by Fosdick and Raftery (2014) for capturing correlation between countries.

We will model the forecast errors as follows:

(7) $$\varepsilon_{t}∼N\left(0,\Sigma_{t}=\sigma_{t}^{'}A\sigma_{t}\right),$$

where ***σ***_***t***_ is a vector consisting of the forecast standard deviations for each region. For Phase II this is the standard deviation of the errors in the double logistic model, and for Phase III it is the standard deviation of the error term in the AR(1) model. In (7), ***A*** is a matrix where each element *A*_*r,s*_ corresponds to the correlation between the model errors of country’s region *r* and *s* over all time periods.

Let *f*_*r,t*_ denote the observed TFR for region *r* at time *t*. We denote by *e*_*r,t*_ the normalized forecast error, namely the forecast error divided by its standard deviation. The normalized forecast error *e*_*r,t*_ is estimated as follows:

- Phase II: For each value *g*_*r,t,i*_ of a double logistic (DL) trajectory *i* and the standard deviation of DL *σ*_*r,i*_ take $d_{r,t,i}=\left(f_{r,t}-g_{r,t,i}\right)/\sigma_{r,i}$. Then *e*_*r,t*_ is the mean of *d*_*r,t,i*_ over *i*.
- Phase III: For each value *h*_*r,t,i*_ in a phase III trajectory *i* and the standard deviation of these trajectories *σ*_*ε*_,_*r,i*_, take the difference $d_{r,t,i}=\left(f_{r,t}-h_{r,t,i}\right)/\sigma_{\varepsilon ,r,i}$. Then, *e*_*r,t*_ is the mean of *d*_*r,t,i*_ over i.

We define the correlation matrix ***A*** as

$$A=\frac{\bar{T}-1}{\bar{T}}\tilde{A}+\frac{1}{2\bar{T}},$$

where $\tilde{A}$ is a truncated correlation matrix made positive definite, and $\bar{T}$ is the average number of time periods per region. Here ***A*** has an approximate Bayesian interpretation as an approximation of the posterior mean with a uniform distribution on [0, 1] for the correlations. Note that ***A*** is positive definite. The appendix contains details of the method as well as other methods for deriving ***A*** that we have experimented with.

## 5. Results

We now compare results from the three methods described in the previous section. All three methods depend on a national BHM simulation. We used a simulation that was used to produce the official UN TFR projections in the WPP 2012 (United Nations 2013). Our version has 2,000 TFR trajectories for each country and was produced using the bayesTFR R package (Ševčíková, Alkema, and Raftery 2011).

For the Scale-AR(1) method, for each region *r*_*c*_ we set the initial scaling factor to $\alpha_{r_{c},P}=f_{r_{c},P}/f_{c,P}$ with *P* being the last observed time period. Then we produced projections of $\alpha_{r_{c},t}$ for *t* > *P* using (3). Finally we applied (1), as in the case of the simple Scale method, using each of the 2,000 TFR trajectories for country *c* as *f*_*c,t,i*_. This yielded 2,000 regional TFR trajectories.

For the one-directional BHM (1d-BHM), we ran the regional BHM while using the country posterior from the national BHM simulation. Then we projected 2,000 regional TFR trajectories using a sample of the regional posterior parameters. We explored two versions of this model, one that accounts for correlation between regions’ error terms and one that does not, the latter denoted by ‘1d-BHM (indep).’

### 5.1 TFR projections

**Figure 5:**
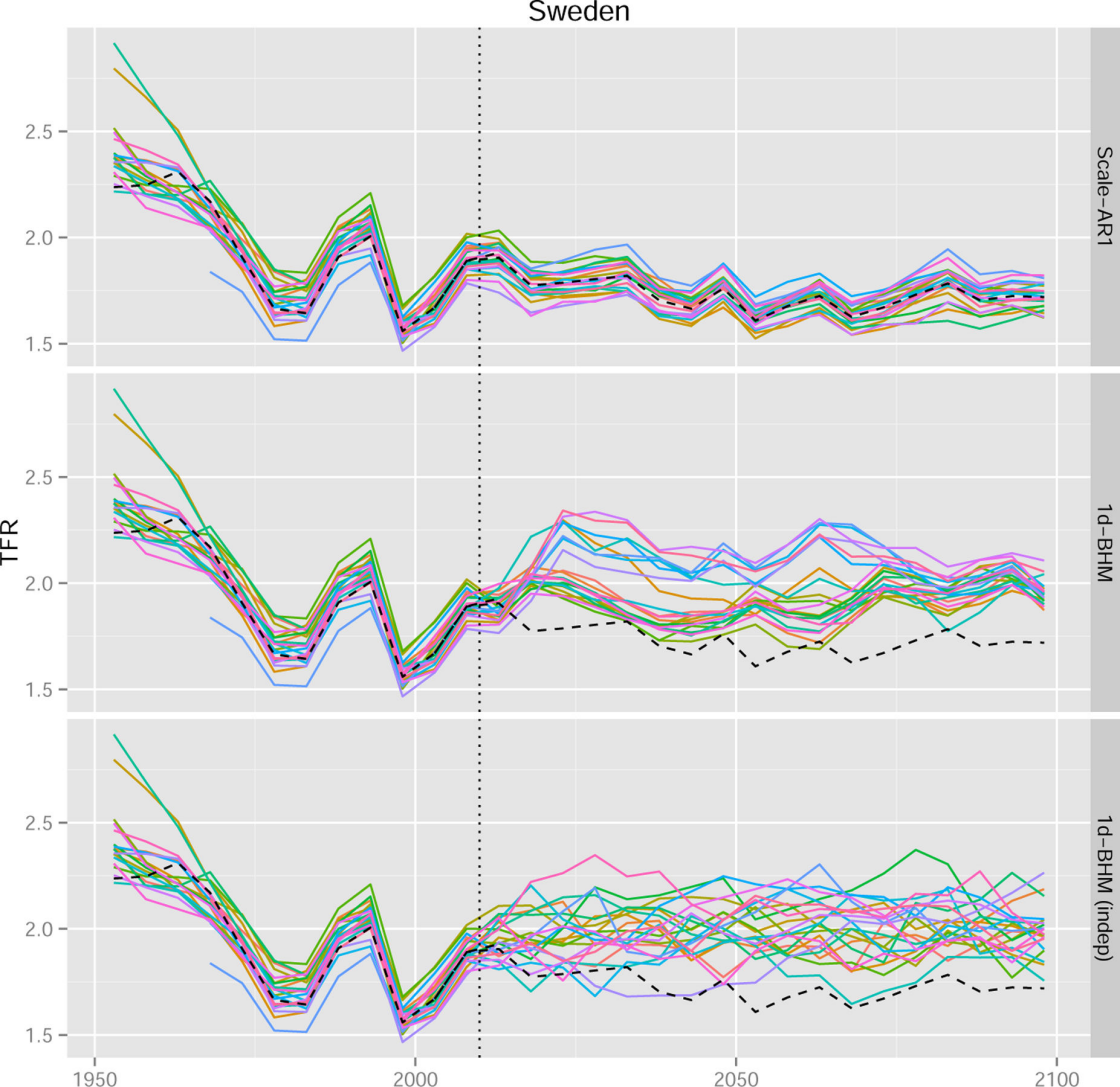
Observed data and one randomly selected projection trajectory for all regions of Sweden *Note*: The projections were obtained via three different methods: Scale-AR(1) (top), the one-directional BHM that accounts for correlation (center), and the one-directional BHM that treats regions independently (bottom). The vertical dotted line marks the last observed time period. The black dashed line marks the corresponding national trajectory.

We are interested in the marginal predictive distribution of future TFR for each region. We are also interested in how reasonable the joint distributions of the trajectories between regions are. Figure 5 shows one randomly selected trajectory for all regions of Sweden for various methods. In the top panel the Scale-AR(1) method was used. It can be seen that all trajectories closely follow the corresponding national trajectory (black dashed line), while allowing for occasional crossovers. This creates a similar pattern to that seen in the observed data (to the left from the dotted vertical line). The simple Scale method (not shown in the figure) yields trajectories perfectly parallel to the national trajectory with no crossovers.

The bottom two panels of Figure 5 show results from the one-directional BHM method. In the middle panel we accounted for correlation between regions, whereas in the bottom panel the regions’ error terms were considered independent. As can be seen, this method does not yield trajectories that closely parallel the national one. Furthermore, if correlation is not taken into account, there are many more crossovers between regions than are typically seen in the past data.

**Figure 6:**
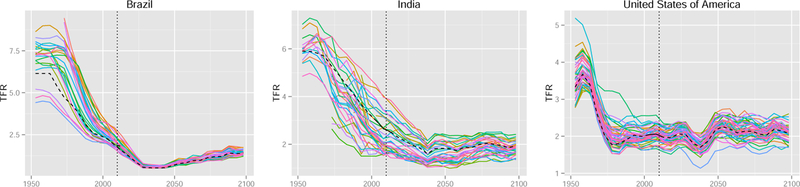
Observed data and one randomly selected projection trajectory for each region, obtained via the Scale-AR(1) method for all regions of Brazil, India, and the United States *Note*: The vertical dotted line marks the last observed time period. The black dashed line marks the corresponding national trajectory.

All the 47 countries in our dataset show the same pattern in terms of the differences between the methods. In Figure 6 we selected three countries for which one trajectory obtained via the Scale-AR(1) method is shown for each region (as in the top panel of Figure 5). As in the case of Sweden, the trajectories are highly correlated and closely follow the national trajectory.

Showing one trajectory for each region, as in Figures 5 and 6, is a good way to see the correlation between regions. It must be the same trajectory, corresponding to the same set of parameters. A mean or median curve, which averages over trajectories, would not convey this information.

**Figure 7:**
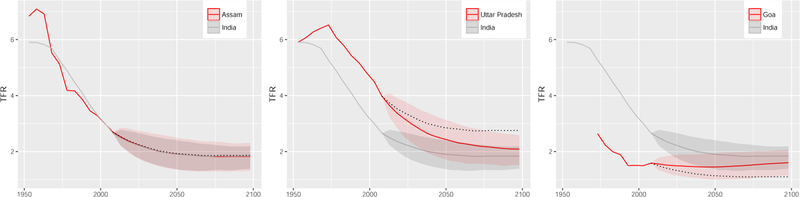
TFR projections for three regions of India *Note*: Observed data and median projections are shown by the red line, and the 80% prediction interval is shown by the red shaded area. National data, projection median, and 80% prediction interval are shown as the gray line and the shaded area, respectively. The dotted line shows the median projection resulting from the simple Scale method.

In Figure 7 we show the predictive median and 80% prediction interval (red) for three regions of India from the Scale-AR(1) method (the corresponding national projection is shown in gray). They represent three different types of regions found across all countries. The first type (in the left panel, Assam, India) is a region with a current TFR that is very close to the national TFR. In such a case, the regional projection mostly overlaps with the national projection, with a slightly larger prediction interval. The black dotted line in the figure shows the median projection resulting from the simple Scale method. This would also be very close to the national median for regions of this type.

Uttar Pradesh in the center is a type of region where current TFR is substantially higher than the national TFR. The underlying AR(1) process causes the median projection of such a region to converge to the national median in the long term, thus decreasing the gap between them. If simple scaling were applied, that gap would remain constant, resulting in much higher projections of TFR for the region.

Finally, Goa on the right, with its current TFR well below the national one, is projected to increase on average, again yielding a smaller gap between the national and regional medians. Here simple scaling results in much lower projections.

Probabilistic projections for the regions of all 47 countries are provided in the supplementary material.

### 5.2 Out-of-sample predictive validation

We validated our methodology via predictive out-of-sample experiments, one for predicting the period 1995–2010 and another one for predicting the period 1990–2010. We first assessed the various methods in terms of average predictive performance over all regions of the 47 countries. To assess their performance for predicting aggregates (and hence, for example, in capturing the between-region correlations), we further assessed the predictions of the average TFR across the regions of each country.

For both time periods considered, we removed the data points that corresponded to the time period to be predicted, reestimated the models, generated probabilistic projections with the various methods, and compared the projections with the observed data points. Thus, for example, for the experiments with 1995–2010, we estimated the model using only the data from 1950–1995, used it to predict the three five-year periods from 1995–2010, and then compared these predictive distributions with what actually happened in 1995–2010.

The results are shown in Table 1 for 1995–2010 and Table 2 for 1990–2010. The measures in the left part of each table (Marginal TFR) were derived by comparing the probabilistic projections of TFR for all regions to their observed values. The quantities in the right part of the tables (Average TFR) were derived by comparing a TFR averaged over all regions of each country with the observed average TFR for each country.

For comparison purposes, we also added the Persistence method, in which the TFR stays at the same level over time, and so the forecast for all future time periods is equal to the last observed value. While this could be viewed as a straw man forecast, persistence forecasts have been found to perform surprisingly well in many forecasting contexts, and so it is worth making this comparison.

In the tables, the mean absolute error (MAE) and the bias and the continuous ranked probability score (CRPS) (Hersbach 2000; Gneiting and Raftery 2007) are reported. The coverages of the 80% and 95% intervals are also reported. The coverage of a prediction interval is defined as the proportion of the time that the truth lies in the interval. We wish the coverage to be close to the nominal level. Thus, for example, ideally the coverage of the 80% interval would be close to 80%.

The appendix gives details of the derivation of these metrics. For MAE and bias, the smaller the absolute value the better. For the two coverage columns an ideal method would match the numbers to the corresponding percentage. The CRPS is a generalization of the MAE to the case of probabilistic forecast. It is a combination of an error-based and a variation-based measure that assesses the difference between the cumulative distribution function of the forecasts and the corresponding cumulative distribution function of the observations. Since it is an overall measure of the quality of the forecast, we give it a high weight when selecting the best method. In this case, a better method corresponds to a larger value of CRPS.

For the marginal TFR, the Scale-AR(1) method performed best in terms of CRPS, MAE, and coverage. The simple Scale method came in second. However, we would not recommend using the simple Scale method because it produces trajectories that are unrealistic in that they do not allow the possibility of crossovers between regions, as mentioned previously. Note that by design, the Scale-AR(1) method yields larger uncertainty than the simple Scale method, which in this case translated to a better coverage and CRPS. The Scale method includes only the uncertainty from the national BHM model, whereas the Scale-AR(1) method has in addition the uncertainty included in the AR(1) process. There was essentially no difference between the 1d-BHM with and without correlation for the marginal TFR. This is expected, as the correlation plays a role only in aggregated indicators.

For the average TFR, the Scale-AR(1) and 1d-BHM had similar performance in terms of CRPS (one was better in Table 1, the other in Table 2). However, Scale-AR(1) had consistently better coverage. Here we see a big difference in coverage between the two versions of 1d-BHM, which does not have good performance if correlation between regions is not taken into account. The good performance of the Scale-AR(1) method suggests that it is accounting adequately for between-region spatial correlation.

## 6. Discussion

We have developed several methods for subnational probabilistic projection of TFR and applied them to data from 47 very diverse countries. All the methods take the national projections from the UN method as their starting point. We found that all the methods we propose performed well in terms of out-of-sample predictive performance and outperformed a simple baseline persistence method.

In the best method, the national trajectories are scaled by a region-specific scaling factor that itself is allowed to vary stochastically but slowly over time. One competing method treats the regions the same way as countries are treated in the UN’s BHM, but this does not yield enough within-country correlation. Even when we introduce additional between-region correlation into this model, it still does not have enough within-country correlation overall.

We have compared several different methods, but there are still others in the literature. Rayer, Smith, and Tayman (2009) considered ex-post assessment of predictive uncertainty for US counties, extending the national ex-post approach of Keyfitz (1981) and Stoto (1983) to the subnational context. Raymer, Abel, and Rogers (2012) used a vector autoregressive model for crude birth rates in three regions of England. While these methods may work well for developed countries that have had low fertility for an extended period, they do not capture the systematic variation in fertility decline rates among higher-fertility countries documented by Alkema et al. (2011), and so they may not be so appropriate for our goal here to develop a method applicable to countries at all levels of the fertility transition.

The extant method closest to our preferred Scale-AR(1) method is one proposed by Wilson (2013), who also proposed scaling a national TFR forecast by a region-specific scale factor that varies according to an AR(1) model and applied it to Sydney, Australia. However, there are several differences between the Scale-AR(1) method we propose here, and Wilson’s approach for TFR. The national TFR forecast used by Wilson is based on an AR(1) process centered around an externally specified main forecast. As discussed, this may not carry over well to higher-fertility countries. Our method, in contrast, is centered around the probabilistic forecast from the UN’s BHM, which is designed to work well for countries at all fertility levels and includes uncertainty about national projections. Also, in our method the model is statistically estimated, while in Wilson’s approach the parameters are adjusted manually.

Our preferred Scale-AR(1) method does not incorporate spatially-indexed between-region correlation. Instead, spatial correlation is modeled by a strong country effect. Our 1-d BHM method does incorporate spatial correlation in the variant that includes between-region correlation estimated from the data (especially methods 8–11 described in the Appendix section on estimating the error correlations). However, this did not allow us to include enough between-region correlation. This may be because within-country correlation seems to be dominated by a strong country effect rather than spatially indexed correlation, as can be seen for example for Sweden in Figure 5. This is also shown by the good calibration of the Scale-AR(1). Thus we feel it is likely that adding additional spatial correlation would not substantially improve fit of the model to the data at hand.

In addition to providing guidance for subnational projections, our results give insight into how subnational fertility evolves in a modern context. They suggest that there is substantial within-country correlation and convergence. This confirms the observations and hypotheses of Watkins (1990, 1991) for Europe to 1960. It further extends them from just Europe to a range of countries from around the world and indicates that, broadly speaking, similar patterns continue to hold a half-century later.

The Scale-AR(1) method is implemented as part of the R package bayesTFR (Ševčíková, Alkema, and Raftery 2011). The help page for the function tfr.predict.subnat gives details on how to use it. An R script to replicate results in this article is available on the journal’s website.

## Figures and Tables

**Table 1:** Out-of-sample validation of probabilistic subnational TFR projections over 1995–2010

	Marginal TFR					Average TFR				
	MAE	Bias	CRPS	80%	95%	MAE	Bias	CRPS	80%	95%
Scale-AR(1)	0.205	−0.088	−0.147	82.0	96.3	0.172	−0.117	−0.127	82.5	95.6
1d-BHM	0.228	−0.067	−0.167	75.1	90.1	0.169	−0.101	−0.123	71.5	89.1
1d-BHM (indep)	0.228	−0.071	−0.167	75.2	89.8	0.169	−0.103	−0.142	38.7	50.4
Scale	0.220	−0.106	−0.156	76.2	92.2	0.182	−0.136	−0.133	78.8	95.6
Persistence	0.365	−0.305	−0.365	–	–	0.334	−0.303	−0.334	–	–

*Note*: MAE is mean absolute error. CRPS is continuous ranked probability score, for which larger is better. The 80% and 95% columns refer to the percentage of the observations that fell within their prediction interval. The marginal TFR was validated on 3199 values; the average TFR was validated on 137 values. The Scale-AR(1) parameters were ϕ = 0.898 and σ = 0.0533.

**Table 2:** Out-of-sample validation of TFR projections over 1990–2010

	Marginal TFR					Average TFR				
	MAE	Bias	CRPS	80%	95%	MAE	Bias	CRPS	80%	95%
Scale-AR(1)	0.323	−0.209	−0.234	70.0	84.8	0.278	−0.192	−0.202	73.3	87.2
1d-BHM	0.344	−0.207	−0.260	64.5	79.1	0.284	−0.187	−0.214	60.0	76.1
1d-BHM (indep)	0.344	−0.209	−0.260	64.6	79.6	0.284	−0.190	−0.242	26.7	41.7
Scale	0.333	−0.230	−0.245	65.6	82.0	0.291	−0.215	−0.210	72.2	87.2
Persistence	0.590	−0.538	−0.590	–	–	0.519	−0.476	−0.522	–	–

*Note*: The marginal TFR was validated on 4144 values; the average TFR was validated on 180 values. The Scale-AR(1) parameters were ϕ = 0.910 and σ = 0.0513.

**Figure A-1:**
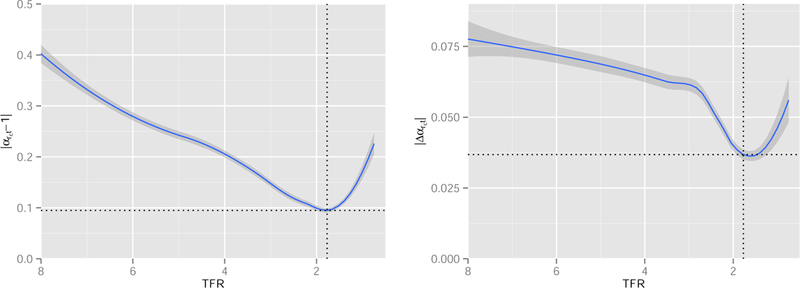
The loess curve for $\left|\alpha_{r_{c},t}-1\right|$ ~ TFR (left panel) and for $\left|\Delta \alpha_{r_{c},t}\right|$ ~ TFR (right panel) Note: The loess curves are based on 9,566 data points.

## Appendix: Methods

### Estimation of the Scale-AR(1) parameters

Here we give details on estimating parameters of the Scale-AR(1) model.

The model is based on an AR(1) process for region-specific scale factors $\alpha_{r_{c},t}$ centered at one, namely

(8) $$\alpha_{r_{c},t}-1=\phi \left(\alpha_{r_{c},t-1}-1\right)+\varepsilon_{r_{c},t},\;\text{with}\;\varepsilon_{r_{c},t}\overset{iid}{\sim }N\left(0,\sigma_{c}^{2}\right).$$

We impose the restriction that the scale factors not diverge indefinitely over time. We implement this by requiring that $\sigma_{c}^{2}$ is such that

(9) $$\underset{t\to \infty }{\lim}\text{Var}\left(\alpha_{r_{c},t}\right)\leq {\text{Var}}_{q\in R_{c}}\left(\alpha_{q,t=P}\right),$$

where *P* denotes the present time period and *R*_*c*_ denotes the set of regions in country *c*. This yields

(10) $$\sigma_{c}^{2}=\min\left\{\sigma^{2},\left(1-\phi^{2}\right){\text{Var}}_{r\in R_{c}}\left(\alpha_{r,t=P}\right)\right\}.$$

We are interested in estimating the country- and region-independent parameters *ϕ* and *σ*. We know from the observed data that the standard deviation of $\alpha_{r_{c},t}$ declines as TFR declines, which is also in line with the theoretical expectations of Watkins (1990, 1991). Thus we need to find asymptotic values for those parameters.

Let $\Delta \alpha_{r_{c},t}$ denote the first order differences over time, namely

(11) $$\Delta \alpha_{r_{c},t}=\alpha_{r_{c},t}-\alpha_{r_{c},t-1}.$$

Then

(12) $$\underset{t\to \infty }{\lim}\text{Var}\left(\alpha_{r_{c},t}\right)=\frac{\sigma^{2}}{1-\phi^{2}}\;\;\text{and}$$

(13) $$\underset{t\to \infty }{\lim}\text{Var}\left(\Delta \alpha_{r_{c},t}\right)=2\left(1-\phi \right)\text{Var}\left(\alpha_{r_{c},t}\right).$$

Equations (12) and (13) imply that

(14) $$\phi =1-\frac{\text{Var}\left(\Delta \alpha_{r_{c},t}\right)}{2\text{Var}\left(\alpha_{r_{c},t}\right)}\;\;\text{and}$$

(15) $$\sigma^{2}=\text{Var}\left(\Delta \alpha_{r_{c},t}\right)-\left(1-\phi^{2}\right)\text{Var}\left(\alpha_{r_{c},t}\right).$$

Assuming a normal distribution of $\alpha_{r_{c},t}$ we can write

(16) $$\text{Var}\left(\alpha_{r_{c},t}\right)=\frac{\pi }{2}{\left(E[|\alpha_{r_{c},t}-1|]\right)}^{2},$$

(17) $$\text{Var}\left(\Delta \alpha_{r_{c},t}\right)=\frac{\pi }{2}{\left(E[|\Delta \alpha_{r_{c},t}|]\right)}^{2}.$$

From the observed data we know that both $\left|\alpha_{r_{c},t}-1\right|$ and $\left|\Delta \alpha_{r_{c},t}\right|$ decline as TFR declines (see Figure A-1). The nonparametrically estimated conditional expectation of $\left|\alpha_{r_{c},t}-1\right|$ given TFR reaches a minimum, as a function of TFR, of 0.09475 at TFR = 1.768, as shown by the dotted lines in Figure A-1. At this level of TFR, the nonparametrically estimated value of $E\left(\left|\Delta \alpha_{r_{c},t}\right|\right)$ is 0.03678, which is close to its minimum. Taking the mean of $\alpha_{r_{c},t}$ to be 1, and using the fact that the standard deviation of a normal random variable is $\sqrt{\pi /2}$ times its mean absolute deviation, we find that

(18) $$\text{SD}\left(\alpha_{r_{c},t}\right)=\sqrt{\frac{\pi }{2}}0.09475=0.11875,$$

(19) $$\text{SD}\left(\Delta \alpha_{r_{c},t}\right)=\sqrt{\frac{\pi }{2}}0.03678=0.04610.$$

Substituting the values from Equations (18) and (19) for $\left(\alpha_{r_{c},t}\right)$ and $\left(\Delta \alpha_{r_{c},t}\right)$ into Equations (14) and (15) gives

(20) $$\begin{array}{l} \phi =0.92464,\\ \sigma =0.04522. \end{array}$$

These are the values we use for our projections.

### Estimating the error correlations

The model errors are defined by

$$\varepsilon_{t}\sim N\left(0,\Sigma_{t}=\sigma_{t}^{'}A\sigma_{t}\right).$$

We have experimented with eleven different ways of estimating the correlations of the errors, which are the elements *A*_*rs*_ of the matrix ***A***. Let *e*_*r*,*t*_ denote the model error of region *r* at time *t*. Let $\tilde{A}$ denote a matrix where each element ${\tilde{A}}_{rs}$ is the empirical correlation between *e*_*r*·_ and *e*_*s*·_ over all time periods *t*, namely

(21) $${\tilde{A}}_{r,s}=\frac{\sum_{T}e_{r,t}e_{s,t}}{\sqrt{\sum_{T}e_{r,t}^{2}}\cdot \sqrt{\sum_{T}e_{s,t}^{2}}}.$$

Furthermore, $\tilde{A}$ truncated at zero will be denoted by ${\tilde{A}}_{\left[\geq 0\right]}$. If positive definiteness is assured, it is denoted by ${\tilde{A}}^{*}$.

We considered the following methods for estimating the matrix ***A***. In the first seven methods, all the within-country correlations are taken to be equal. The estimator of ***A*** is denoted by $\hat{A}$. In all cases, ${\hat{A}}_{r,r}=1$ for all *r*, so in what follows, ${\hat{A}}_{r,s}$ refers

to the cases where *r* ≠ *s*.

1. ${\hat{A}}_{r,s}=\text{mean}\{a_{ij}\in {\tilde{A}}_{\left[\geq 0\right]}\;\text{and}\;i\neq j\}$ for all *r* ≠ *s*.
2. ${\hat{A}}_{r,s}=\text{median}\{a_{ij}\in {\tilde{A}}_{\left[\geq 0\right]}\;\text{and}\;i\neq j\}$ for all *r* ≠ *s*.
3. ${\hat{A}}_{r,s}$ is the Bayesian posterior mean of the intraclass correlation coefficient.
4. ${\hat{A}}_{r,s}$ is the Bayesian posterior mode of the intraclass correlation coefficient.
5. Similar to 3, but with errors divided by $\sqrt{1/n\sum_{r,t}e_{r,t}^{2}}$ with *n* being the number of available errors.
6. Similar to 4, but with errors divided by $\sqrt{1/n\sum_{r,t}e_{r,t}^{2}}$ with *n* being the number of available errors.
7. ${\hat{A}}_{r,s}=B/C$ for all *r ≠ s*, where
  $$\begin{array}{l} B=\frac{1}{N_{b}}\sum_{t=1}^{T}\sum_{i=1}^{R-1}\sum_{j=i+1}^{R}e_{i,t}\cdot e_{j,t},\\ C=\frac{1}{N_{c}}\sum_{t=1}^{T}\sum_{i=1}^{R}e_{i,t}^{2}, \end{array}$$
  *N*_*b*_ and *N*_*c*_ the number of terms in the corresponding sum that are not missing, and *R* is the number of regions.
8. The estimator of ***A*** is an approximation to the elementwise posterior median with uniform prior *U* [0, 1], namely:
  $$\hat{A}=\frac{\bar{T}-1}{\bar{T}}{\tilde{A}}^{*}{}_{\left[\geq 0\right]}+\frac{1}{2\bar{T}},$$
  where $\bar{T}$ is the average number of time periods per region. It is a weighted average of the prior mean and the data. Note that this is our chosen method. We will now show that if ${\tilde{A}}^{*}{}_{\left[\geq 0\right]}$ is positive definite, then ***A*** is also positive definite. We can write
  (22) $$\hat{A}=\frac{T-1}{T}B+\frac{1}{2T}J,$$
  where ***B*** is positive definite and ***J*** is the matrix all of whose entries are 1. Now $\hat{A}$ is positive definite if and only if $x^{\prime }\hat{A}x>0$ for all *x ≠* 0. Now
  (23) $$x^{\prime }\hat{A}x=\frac{T-1}{T}x^{\prime }Bx+\frac{1}{2T}x^{\prime }Jx.$$
  The first term on the right-hand side of (23) is positive by definition, since ***B*** is positive definitive. The second term is non-negative:
  (24) $$x^{\prime }Jx={\left(\sum_{i=1}^{n}x_{i}\right)}^{2}\geq 0.$$
  Thus (23) is positive and so $\hat{A}$ is positive definite.
9. Similar to 8, but with elements of $\tilde{A}$ computed as
  $${\tilde{A}}_{r,s}=\frac{1/T\sum_{T}e_{r,t}e_{s,t}}{\sqrt{1/T\sum_{T}e_{r,t}^{2}}\cdot \sqrt{1/T\sum_{T}e_{s,t}^{2}}}.$$
10. Bayesian method introduced by Fosdick and Raftery (2014): First, standardize *e*_*r,t*_ by dividing the errors by $\sqrt{1/n\sum_{r,t}e_{r,t}^{2}}$ with n being the number of available errors. Then the elements of the estimated correlation matrix $\hat{A}$ are given as
  $${\hat{A}}_{r,s}=\frac{\int_{0}^{1}\rho {\left(\frac{1}{\sqrt{1-\rho^{2}}}\right)}^{T}\exp\left[-\frac{1}{2\left(1-\rho^{2}\right)}\left[SS_{r}-2\rho SS_{r,s}+SS_{s}\right]\right]d\rho }{\int_{0}^{1}{\left(\frac{1}{\sqrt{1-\rho^{2}}}\right)}^{T}\exp\left[-\frac{1}{2\left(1-\rho^{2}\right)}\left[SS_{r}-2\rho SS_{r,s}+SS_{s}\right]\right]d\rho },$$
  where $SS_{r}=\sum_{T}e_{r,t}^{2}$, $SS_{s}=\sum_{T}e_{s,t}^{2}$, and $SS_{r,s}=\sum_{T}e_{r,t}e_{s,t}$. Note that we are summing only over those time periods for which both countries, *r* and *s*, have errors available.
11. Similar to 10, but using (*T* + 1) instead of *T* in both the nominator and the denominator. This corresponds to the arcsin prior in Fosdick and Raftery (2014). Note that a version of this method was tested where the errors were not standardized, but it performed less well, producing smaller correlations.

Fosdick and Raftery (2014) found that correlations between countries were quite different for high and low TFR values. In light of this, we estimated two separate correlation matrices, one for the cases where the country had overall TFR 5 or above and the other when the TFR was below 5.

The estimated correlation matrices resulting from methods 1–7 have the same value for all off-diagonal elements. The elements of matrices resulting from methods 8–11 differ from one another. In the latter case, all nondefined elements are set to the mean of the off-diagonal elements.

### Out of sample validation measures

This section provides detailed definitions for our out-of-sample validation measures. We denote by *C* the number of countries in our dataset, by *R*_*c*_ the number of regions for country *c*, by *R* the total number of regions, so that $R=\sum_{c=1}^{C}R_{c}$, and by *T* the number of time periods over which we validate. Furthermore, $f_{r_{c},t}$ denotes the observed TFR, and ${\hat{f}}_{r_{c},t}$ denotes the point projection of the TFR (median of the predictive distribution), respectively, for region *r* of country *c* at time *t*.

The mean absolute error, MAE, is given by

(25) $$\text{MAE}=\frac{1}{RT}\sum_{c=1}^{C}\sum_{r=1}^{R_{c}}\sum_{t=1}^{T}\left|f_{r_{c},t}-{\hat{f}}_{r_{c},t}\right|.$$

The bias is given by

(26) $$\text{bias}=\frac{1}{RT}\sum_{c=1}^{C}\sum_{r=1}^{R_{c}}\sum_{t=1}^{T}\left(f_{r_{c},t}-{\hat{f}}_{r_{c},t}\right).$$

The continuous ranked probability score (CRPS) is an overall measure of the quality of a probabilistic forecast. If we are predicting the quantity *X* (here the TFR), and produce the predictive distribution *F* , and observe a value *x*, then the CRPS is defined by:

$$\text{CRPS}\left(F;x\right)=\frac{1}{2}E_{F}\left|X^{\prime }-X^{\prime \prime }\right|-E_{F}\left|X-x\right|,$$

where *X′* and *X*″ are independent copies of a random variable with the distribution *F* (Gneiting and Raftery 2007, Eq. 21). We average the resulting values of CRPS across observations. Note that for the persistence method, the first part of the equation is zero. It is not simple to calculate the expectation, *E*_*F*_ , under the distribution *F* analytically, so we did it by simulation. To obtain the expectation *E*_*F*_ , we sampled 5,000 values at random from the distribution *F* and took the average of the corresponding predictands.

So far we have compared the methods for the TFR for all regions and for the average TFR for a country, taking the unweighted average over all regions in the country (Tables 1 and 2). Here, we add a comparison of the various correlation methods discussed above for the average TFR (Tables A-1 and A-2). The first row shows results when no correlation is taken into account. The number in parentheses of the following rows corresponds to the numbering of the eleven methods in the Appendix. In our study, we used method 8, which performed at least as well as the other variants. The different approaches that used correlation performed about equally well. Tables 1 and 2 show that the Scale-AR(1) method was better calibrated than the others in the sense that its prediction intervals had coverage much closer to the desired nominal level than the other methods.

**Table A-1:** Out-of-sample validation of average TFR projections over 1995–2010, validated on 137 values

	MAE	Bias	CRPS	80%	95%
1d-BHM (indep.)	0.169	−0.103	−0.142	38.7	50.4
1d-BHM (1.)	0.170	−0.101	−0.124	69.3	89.1
1d-BHM (2.)	0.167	−0.102	−0.122	74.5	89.8
1d-BHM (3.)	0.168	−0.104	−0.127	62.0	80.3
1d-BHM (4.)	0.167	−0.105	−0.127	62.8	81.0
1d-BHM (5.)	0.167	−0.106	−0.124	69.3	84.7
1d-BHM (6.)	0.167	−0.106	−0.125	68.6	83.9
1d-BHM (7.)	0.168	−0.104	−0.125	65.7	83.2
1d-BHM (8.)	0.169	−0.101	−0.123	71.5	89.1
1d-BHM (9.)	0.169	−0.100	−0.123	70.1	89.1
1d-BHM (10.)	0.168	−0.102	−0.123	70.8	89.1
1d-BHM (11.)	0.167	−0.103	−0.122	71.5	89.8

Scale-AR(1)	0.172	−0.117	−0.127	82.5	95.6
Scale	0.182	−0.136	−0.133	78.8	95.6
Persistence	0.334	−0.303	−0.334	–	–

**Table A-2:** Out-of-sample validation of average TFR projections over 1990–2010, validated on 180 values

	MAE	Bias	CRPS	80%	95%
1d-BHM (indep.)	0.284	−0.190	−0.242	26.7	41.7
1d-BHM (1.)	0.284	−0.187	−0.213	60.0	77.2
1d-BHM (2.)	0.279	−0.190	−0.210	63.3	78.3
1d-BHM (3.)	0.284	−0.192	−0.217	55.6	71.1
1d-BHM (4.)	0.283	−0.192	−0.216	55.6	70.0
1d-BHM (5.)	0.284	−0.194	−0.215	57.8	76.1
1d-BHM (6.)	0.284	−0.194	−0.216	57.8	74.4
1d-BHM (7.)	0.284	−0.191	−0.214	56.7	72.2
1d-BHM (8.)	0.284	−0.187	−0.214	60.0	76.1
1d-BHM (9.)	0.284	−0.187	−0.213	60.0	76.1
1d-BHM (10.)	0.285	−0.188	−0.215	60.0	76.7
1d-BHM (11.)	0.284	−0.189	−0.214	61.1	77.2

Scale-AR(1)	0.278	−0.192	−0.202	73.3	87.2
Scale	0.291	−0.215	−0.210	72.2	87.2
Persistence	0.519	−0.476	−0.522	–	–

**Table A-3:** Sources of the data used in the study

Country	Geographic units	Units	Obs.	NUTS or eqv.	Subnational TFR data source
Argentina	Provinces (Jurid.)	24	321	2	Pantelides (2006, 1989); INDEC (2012).
Australia	States, Territories	8	104	1	Australian Bureau of Statistics (2008, 2010).
Austria	States	9	90	2	Statistics Austria (2012).
Belgium	Regions	3	24	1	Statistics Belgium (2012).
Brazil	States	27	332	2	IBGE (2012); ECLAC and CELADE (2012a).
Bulgaria	Regions	6	24	2	Eurostat (2012); National Statistical Institute of Bulgaria.
Canada	Provinces-	13	189	2	Statistics Canada (2012).
Chile	Territories Regions	15	136	1	ECLAC and CELADE (2012b); INE Chile (2012).
China	Provinces	31	329	2	1982–2010 censuses in Yao (1995); National Bureau of Statistics of China and East-West Centre (2007); PCO and DPS (1985); National Bureau of Statistics of China (1993, 2002, 2007, 2012).
Costa Rica	Provinces	7	77	1	Rosero-Bixby (2012). The estimates are based in the Vital Statistics on Births, published by the Instituto Nacional de Estadística y Censos (INEC) and interpolation of the female population from the 1950, 1963, 1973, 1984, 2000, and 2011 censuses. Both births and population were corrected following Brenes (2012).
Cuba	Provinces	15	120	2	Tasa de Natalidad (por 1000 habitantes) según provincia de residencia, período 1970–2002 in Oficina Nacional de Estadísticas de Cuba (2005a); Tasas del movimiento natural por provincias in Oficina Nacional de Estadísticas de Cuba (2005b).
Czech Republic	Regions	14	84	3	Czech Statistical Office (2012a, 2012b, 2012c).
Denmark	Counties	16	96	3	Statistics Denmark (2012).
Ecuador	Provinces	22	198	2	ECLAC and CELADE (2012c).
Estonia	Counties	16	64	4	Statistics Estonia (2012).
Finland	Regions	19	95	3	Statistics Finland (2012)
France	Departements	96	1042	3	INSEE (2006, 2012); Lincot and Lutinier (2006).
Germany	States	16	142	1	Germany Federal Statistical Office (2012); Klüsener (2012).
Greece	Regions	13	52	2	Eurostat (2012).
Hungary	Regions	7	28	2	Eurostat (2012).
India	States	33	510	2	Ram and Ram (2009); Rele (1987); Office of the Registrar General and Census Commissioner.
Indonesia	Provinces	33	236	2	ESCAP (1987); Badan Pusat Statistik (2010).
Iran	Provinces	26	152	2	Statistical Centre of Iran (2001); Abbasi-Shavazi and McDonald (2005).
Italy	Regions	21	242	2	Eurostat (2012); ISTAT (2012).
Japan	Prefectures	47	752	2	National Institute of Population and Social Security Research of Japan (2004); Statistics of Japan (2012).
Korea	Provinces	16	135	1	Rele (1988); Statistics Korea (2012).
Mexico	States	32	256	2	Partida Bush (2008); ECLAC and CELADE (2012d).
Norway	Counties	19	171	4	Statistics Norway (2012).
Panama	Provinces	12	90	1	ECLAC and CELADE (2012e).
Paraguay	Departments	18	144	2	ECLAC and CELADE (2012f).
Poland	Provinces	16	64	2	Central Statistical Office of Poland (2012); Eurostat (2012).
Portugal	Regions	7	28	2	Eurostat (2012); Statistics Portugal (2012).
Romania	Counties	42	398	3	National Institute of Statistics of Romania (2006, 2012).
Russia	Regions	82	525	2	Russia in 1946–1958 in Andreev, Darsky, and Kharkova (1998); Russian Federal State Statistics Service (2012).
Serbia	Regions	3	36	2	Statistical Office of the Republic of Serbia (2012).
Slovakia	Regions	8	24	3	Statistical Office of the Slovak Republic (2012).
Slovenia	Macroregions	2	8	2	Eurostat (2012); Statistical Office of the Republic of Slovenia (2012).
Spain	Autonomous	17	153	2	INE (2012).
Sweden	communities Counties	21	567	3	Statistics Sweden (1999, 2012a, 2012b).
Switzerland	Cantons	26	556	3	Wanner (2000); Office Fédéral de la Statistique (2012).
Thailand	Regions	5	44	1	Pejaranonda (1985); National Statistics Office of Thailand (1997); UNFPA (2011).
Turkey	Provinces	81	810	3	Shorter, Macura, and the Panel on Turkey (1982); Provincial estimates courtesy of Prof. Sinan Turkyilmaz (Hacettepe University Institute of Population Studies) for 1998, 2003, and 2008 TDHS.
Ukraine	Regions	27	133	2	State Statistics Service of Ukraine (2012).
United	Countries	4	34	1	ONS (2012).
Kingdom Uruguay	Departments	19	171	2	ECLAC and CELADE (2012g).
United States	States	51	1184	2	US Census Office (1902); Linder and Grover (1947); NCHS (1977, no year).
Venezuela	States	25	196	2	ECLAC and CELADE (2012h).

## References

[R1] Abbasi-Shavazi M and McDonald P (2005). National and provincial-level fertility trends in Iran, 1972–2000 Canberra: Australian National University (Working Papers in Demography No. 94).

[R2] Alkema L, Raftery AE, Gerland P, Clark SJ, Pelletier F, Buettner T, and Heilig GK (2011). Probabilistic projections of the total fertility rate for all countries. Demography 48(3): 815–839. doi:10.1007/s13524-011-0040-5. 21748544 PMC3367999

[R3] Andreev E (2012). Age-specific fertility rates for Russia in 1959–2010 and regions in 1989–2010

[R4] Andreev E, Darsky L, and Kharkova T (1998). Demographic history of Russia: 1927– 1959 Moscow: Informatika.

[R5] Arokiasmy P and Goli S (2012). Fertility convergence in the Indian states: An assessment of changes in averages and inequalities in fertility. Genus 68: 65–88.

[R6] Australian Bureau of Statistics (2008). 3105.0.65.001: Australian Historial Population Statistics, 2008. [electronic resource] Canberra: Australian Bureau of Statistics http://www.abs.gov.au/AUSSTATS/abs.nsf/Lookup/3105.0.65.001Main+Features12008?OpenDocument.

[R7] Australian Bureau of Statistics (2010). 3101.0: Australian Demographic Statistics, June 2010. [electronic resource] Canberra: Australian Bureau of Statistics.

[R8] Badan Pusat Statistik (2010). Fertilitas penduduk Indonesia: Hasil sensus penduduk 2010 Jakarta: Badan Pusat Statistik.

[R9] Basten S, Huinink J, and Klüsener S (2012). Spatial variation of sub-national fertility trends in Austria, Germany and Switzerland. Comparative Population Studies 36(2–3): 12–52. doi:10.4232/10.CPoS-2011-08en.

[R10] Borges GM (2015). Subnational fertility projections in Brazil: A Bayesian probabilistic approach application. Poster presented at the Annual Meeting of the Population Association of America, San Diego, USA, April 30–May 2, 2015.

[R11] Brenes G (2012). Evaluation of the 2011 census and demographic estimates for the period 1950–2011 [unpublished manuscript] San Jose: Centro Centroamerican de Poblacion, Universidad de Costa Rica.

[R12] Calhoun C (1993). Book review: From provinces into nations: Demographic integration in Western Europe, 1870–1960, by Susan Cotts Watkins. Journal of Modern History 65(3): 597–599. doi:10.1086/244688.

[R13] Central Statistical Office of Poland (2012). Total fertility rate by voivodships in Poland in 1990, 1995 and 1998–2011 Warsaw: Central Statistical Office of Poland.

[R14] Chamie J (1994). Trends, variations, and contradictions in national policies to influence fertility. Population and Development Review 20(Supplement): 37–50. doi:10.2307/2807938.

[R15] Czech Statistical Office (2012a). Demographic yearbooks 1982–1990. [electronic resource] Prague: Czech Statistical Office http://www.czso.cz/csu/redakce.nsf/i/casovaradademografie.

[R16] Czech Statistical Office (2012b). Fertility in Czech Republic since 1991–2006: National and regional level, NUTS3 and LAU1 Prague: Czech Statistical Office.

[R17] Czech Statistical Office (2012c). Demographic yearbook of the regions of the Czech Republic 2002–2011. [electronic resource] Prague: Czech Statistical Office http://www.czso.cz/csu/2012edicniplan.nsf/engpubl/4027-12-eng_r2_012.

[R18] de Beer J and Deerenberg I (2007). An explanatory model for projecting regional fertility differences in the Netherlands. Population Research and Policy Review 26(5).

[R19] Dorius SF (2008). Global demographic convergence? A reconsideration of changing intercountry inequality in fertility. Population and Development Review 34(3): 519–537. doi:10.1111/j.1728-4457.2008.00235.x.

[R20] ECLAC and CELADE (2012a). Redatam+SP custom tabulations 10/26/2012 of Estimación Indirecta de la Fecundidad for Chile: Censo de Población y Vivienda 1980–2002 by regions. [electronic resource] Santiago de Chile: Economic Commission for Latin America and the Caribbean and the Latin American and Caribbean Demographic Centre http://www.redatam.org/redatam/en/.

[R21] ECLAC and CELADE (2012b). Redatam+SP custom tabulations 10/26/2012 of Estimación Indirecta de la Fecundidad for Brasil states: Censo de Población y Vivienda 1980–2010 by states. [electronic resource] Santiago de Chile: Economic Commission for Latin America and the Caribbean and the Latin American and Caribbean Demographic Centre http://www.redatam.org/redatam/en/.

[R22] ECLAC and CELADE (2012c). Redatam+SP custom tabulations 10/26/2012 of Estimación Indirecta de la Fecundidad for Ecuador: Censo de Población y Vivienda 1982–2010 by provinces. [electronic resource] Santiago de Chile: Economic Commission for Latin America and the Caribbean and the Latin American and Caribbean Demographic Centre http://www.redatam.org/redatam/en/.

[R23] ECLAC and CELADE (2012d). Redatam+SP custom tabulations 10/26/2012 of Estimación Indirecta de la Fecundidad for México: Censo de Población y Vivienda 2010 by states. [electronic resource] Santiago de Chile: Economic Commission for Latin America and the Caribbean and the Latin American and Caribbean Demographic Centre http://www.redatam.org/redatam/en/.

[R24] ECLAC and CELADE (2012e). Redatam+SP custom tabulations 10/26/2012 of Estimación Indirecta de la Fecundidad for Panama: Censo de Población y Vivienda 1990–2010 by provinces. [electronic resource] Santiago de Chile: Economic Commission for Latin America and the Caribbean and the Latin American and Caribbean Demographic Centre http://www.redatam.org/redatam/en/.

[R25] ECLAC and CELADE (2012f). Redatam+SP custom tabulations 10/26/2012 of Estimación Indirecta de la Fecundidad for Paraguay: Censo de Población y Vivienda 1982–2002 by departments. [electronic resource] Santiago de Chile: Economic Commission for Latin America and the Caribbean and the Latin American and Caribbean Demographic Centre. http://www.redatam.org/redatam/en/.

[R26] ECLAC and CELADE (2012g). Redatam+SP custom tabulations 10/26/2012 of Estimación Indirecta de la Fecundidad for Uruguay: Censo de Población y Vivienda 1985–2011 by departments. [electronic resource] Santiago de Chile: Economic Commission for Latin America and the Caribbean and the Latin American and Caribbean Demographic Centre http://www.redatam.org/redatam/en/.

[R27] ECLAC and CELADE (2012h). Redatam+SP custom tabulations 10/26/2012 of Estimación Indirecta de la Fecundidad for Venezuela: Censo de Población y Vivienda 1990–2011 by states. [electronic resource] Santiago de Chile: Economic Commission for Latin America and the Caribbean and the Latin American and Caribbean Demographic Centre http://www.redatam.org/redatam/en/.

[R28] ESCAP (1987). Levels and trends of fertility in Indonesia based on the 1971 and 1980 population censuses: A study of regional differentials Bangkok: United Nations, Economic and Social Commission for Asia and the Pacific.

[R29] Eurostat (2012). Fertility rates by age and NUTS 2 region. [electronic resource] Luxembourg: Eurostat http://ec.europa.eu/eurostat/web/products-datasets/product?code=demorfrate2.

[R30] Fosdick BK and Raftery AE (2014). Regional probabilistic fertility forecasting by modeling between-country correlations. Demographic Research 30(35): 1011–1034. doi:10.4054/DemRes.2014.30.35. 25242889 PMC4169201

[R31] Gauthier AH (2007). The impact of family policies on fertility in industrialized countries: A review of the literature. Population Research and Policy Review 26(3): 323–346. doi:10.1007/s11113-007-9033-x.

[R32] Germany Federal Statistical Office (2012). Zusammengefasste Geburtenziffer (total fertility rate) auf Bundeslandebene (Einheit: Kinder je Frau) for 1990–2010 by Bundesland: Courtesy of Frank Swiaczny Wiesbaden: Germany Federal Statistical Office.

[R33] Gneiting T and Raftery AE (2007). Strictly proper scoring rules, prediction, and estimation. Journal of the American Statistical Association 102(477): 359–378. doi:10.1198/016214506000001437.

[R34] Gullickson A (2001). Multiregional probabilistic forecasting [unpublished manuscript] Berkeley: University of California http://u.demog.berkeley.edu/aarong/PAPERS/gullickiiasastochmig.pdf.

[R35] Gullickson A and Moen J (2001). The use of stochastic methods in local area population forecasts [unpublished manuscript] Berkeley: University of California http://www.demog.berkeley.edu/aarong/PAPERS/gullickmoenpaa2001stochminn.pdf.

[R36] Hersbach H (2000). Decomposition of the continuous ranked probability score for ensemble prediction systems. Weather and Forecasting 15: 559–570. doi:10.1175/1520-0434(2000)015¡0559:DOTCRP¿2.0.CO;2.

[R37] Hirschman C (1994). Why fertility changes. Annual Review of Sociology 20(1): 203–233. doi:10.1146/annurev.soc.20.1.203. 12318868

[R38] IBGE (2012). Tfr estimates by Brazilian states for 1940–2000 from census data and for 2001–2009 from Pesquisa Nacional por Amostra de Domicílios (PNAD) survey data (TFR BY Brazil 1940–2009.xlsx). [electronic resource] Rio de Janeiro: Instituto Brasileiro de Geografia e Estatística https://www.ibge.gov.br/english/.

[R39] INDEC (2012). Dinámica y estructura de la Población: Tasa bruta de natalidad por provincia: Años 1980–2009. [electronic resource] Canberra: Instituto Nacional de Estadística y Censo https://web.archive.org/web/20131114005930/http://www.indec.gov.ar/principal.asp?idtema=7924.

[R40] INE (2012). Demografía y Población. [electronic resource] Madrid: Instituto Nacional de Estadística http://www.ine.es/dyngs/INEbase/es/categoria.htm?c=EstadisticaPcid=1254734710990.

[R41] INE Chile (2012). TFR estimates for 1997–2010 by Chilean regions. [electronic resource] Santiago de Chile: Instituto Nacional de Estadísticas http://palma.ine.cl/demografia/SELECCIONINDICADORES.aspx.

[R42] INSEE (2006). Données de démographie régionale 1954 á 1999: CD-ROM Paris: Institut National de la Statistique et des Études Économiques.

[R43] INSEE (2012). 1990–2009 statistiques de l’état civil et estimations de population: Tableau p3d: Indicateurs généraux de population par département et région. [electronic resource] Paris: Institut National de la Statistique et des Études Économiques https://www.insee.fr/fr/statistiques/fichier/2521239/sd2010p3dfe.xls.

[R44] ISTAT (2012). Serie storiche: Tavola 2.7.1. [electronic resource] Rome: Italian National Institute of Statistics http://seriestoriche.istat.it/index.php?id=1nocache=1L=1.

[R45] Kalwij A (2010). The impact of family policy expenditure on fertility in Western Europe. Demography 47(2): 503–519. doi:10.1353/dem.0.0104. 20608108 PMC3000017

[R46] Keyfitz N (1981). The limits of population forecasting. Population and Development Review 7(4): 579–593. doi:10.2307/1972799.

[R47] Klüsener S (2012). TFR by Laender for selected years covering 1950–2008: Courtesy of Frank Swiaczny Rostock: Max Planck Institute for Demographic Research.

[R48] Klüsener S, Perelli-Harris B, and Gassen NS (2013). Spatial aspects of the rise of nonmarital fertility across Europe since 1960: The role of states and regions in shaping patterns of change. European Journal of Population 29(2): 137–165. doi:10.1007/s10680-012-9278-x.

[R49] Lee R, Miller T, and Edwards RD (2003). The growth and aging of California’s population: Demographic and fiscal projections, characteristics and service needs Berkeley: University of California (CEDA Papers, Special Report).

[R50] Lincot L and Lutinier B (2006). Les evolutions demographiques departementales et regionales entre 1975 et 1994 Paris: Institut National de la Statistique et des Études Économiques.

[R51] Linder F and Grover R (1947). Vital statistics rates in the United States 1900–1940 Washington, D.C: National Office of Vital Statistics.

[R52] Luci-Greulich A and Thévenon O (2013). The impact of family policies on fertility trends in developed countries. European Journal of Population 29(4): 387–416. doi:10.1007/s10680-013-9295-4.

[R53] National Bureau of Statistics of China (1993). Data of the 1990 Population Census of China Beijing: China Statistical Publishing House.

[R54] National Bureau of Statistics of China (2002). Tabulation on the 2000 Population Census of China Beijing: China Statistical Publishing House.

[R55] National Bureau of Statistics of China (2007). Results of the 2005 national 1% population sample census Beijing: China Statistical Publishing House.

[R56] National Bureau of Statistics of China (2012). Tabulation on the 2010 Population Census of the People’s Republic of China Beijing: China Statistical Publishing House.

[R57] National Bureau of Statistics of China and East-West Centre (2007). Fertility estimates for provinces of China: 1975–2000 Beijing: China Statistical Publishing House.

[R58] National Institute of Population and Social Security Research of Japan (2004). Demographic sourcebook: Vital statistics for 1930–2003 by prefectures. [electronic resource] Tokyo: Ministry of Health, Labour and Welfare http://www8.cao.go.jp/shoushi/whitepaper/w-2004/html-h/html/g3400000.html.

[R59] National Institute of Statistics of Romania (2006). Demographic yearbook 2006: Table 7: Live births and live-birth rate by counties, 1966–2005 Bucharest: National Institute of Statistics.

[R60] National Institute of Statistics of Romania (2012). Evenimente demografice în anul 1993–2010: Indicatorul conjunctural al fertilitatii by counties: Table 23 for 1993, table 22 for 1994, tables 34 for 1995–2010). [electronic resource] Bucharest: National Institute of Statistics http://www.insse.ro.

[R61] National Statistical Institute of Bulgaria (no year). Population: Demography, migration and projections). [electronic resource] Sofia: National Statistical Institute http://www.nsi.bg/en/content/6593/population-demography-migration-and-projections.

[R62] National Statistics Office of Thailand (1997). Report on the 1995–1996 Survey of population change Bangkok: National Statistics Office.

[R63] NCHS (1977). Birth and fertility rates for states and metropolitan areas Hyattsville: National Center for Health Statistics (DHEW Publication No. (HRA) 78–1905).

[R64] NCHS (no year). Vital statistics data. [electronic resource] Hyattsville: National Center for Health Statistics https://www.cdc.gov/nchs/dataaccess/vitalstatsonline.htm.

[R65] NISRA (2014). Population projections for areas within Northern Ireland: 2014-based Belfast: Northern Ireland Statistics and Research Agency (Methodology Paper). https://www.nisra.gov.uk/sites/nisra.gov.uk/files/publications/SNPP14-Methodology.pdf.

[R66] O’Connell M (1981). Regional fertility patterns in the United States: Convergence or divergence? International Regional Science Review 6(1): 1–14. doi:10.1177/016001768100600101. 12312179

[R67] Office Fédéral de la Statistique (2012). Indicateur conjoncturel de fécondité selon le canton Neuchâtel: Office fédéral de la statistique. https://www.bfs.admin.ch/bfs/de/home/statistiken/bevoelkerung.assetdetail.3442623.html.

[R68] Office of the Registrar General and Census Commissioner (no year). 1971–2010 sample registration system. [electronic resource] New Delhi: Office of the Registrar General and Census Commissioner http://www.censusindia.gov.in/2011-Common/SampleRegistrationSystem.html.

[R69] Oficina Nacional de Estadísticas de Cuba (2005a). Series demográficas 1982–2002: Tomo I Havana: Oficina Nacional de Estadísticas de Cuba.

[R70] Oficina Nacional de Estadísticas de Cuba (2005b). anuario demográfico de Cuba 2005– 2011. [electronic resource] Havana: Oficina Nacional de Estadísticas de Cuba http://www.one.cu/PublicacionesDigitales/PublicacionesDigitales.asp?cod=A.

[R71] ONS (2012). Total fertility rates for the UK and its constituent countries for 1938–2010. [electronic resource] London: Office for National Statistics, Vital Statistics Outputs Branch http://www.ons.gov.uk/ons/datasets-and-tables/index.html.

[R72] Pantelides EA (1989). La fecundidad adolescente en la Argentina al comienzo del Siglo XXI Buenos Aires: Centro de Estudios de Población.

[R73] Pantelides EA (2006). La transicion de la fecundidad en la Argentina 1869–1947 Buenos Aires: Centro de Estudios de Población.

[R74] Partida Bush V (2008). Proyecciones de la Población de méxico, de las entidades federativas, de los municipios y de las localidades 2005–2050 Mexico City: Consejo Nacional de Población (Documento metodológico).

[R75] PCO and DPS (1985). 1982 Population Census of China: Results of computer tabulation Beijing: China Statistical Publishing House.

[R76] Pejaranonda C (1985). Declines in fertility by district in Thailand: An analysis of the 1980 census Bangkok: United Nations, Economic and Social Commission for Asia and the Pacific.

[R77] Raftery AE, Alkema L, and Gerland P (2014). Bayesian population projections for the United Nations. Statistical Science 29(1): 58–68. doi:10.1214/13-STS419. 25324591 PMC4196216

[R78] Raftery AE, Li N, Ševčíková H, Gerland P, and Heilig GK (2012). Bayesian probabilistic population projections for all countries. Proceedings of the National Academy of Sciences 109(35): 13915–13921. doi:10.1073/pnas.1211452109. PMC3435191 22908249

[R79] Ram U and Ram F (2009). Fertility in India: Policy issues and program challenges. In: Singh K, Yadava R, and Pandey A (eds.). Population, poverty and health: Analytical approaches New Delhi: Hindustan: 45–67.

[R80] Rayer S, Smith SK, and Tayman J (2009). Empirical prediction intervals for county population forecasts. Population Research and Policy Review 28: 773–793. doi:10.1007/s11113-009-9128-7. 19936030 PMC2778678

[R81] Raymer J, Abel GJ, and Rogers A (2012). Does specification matter? Experiments with simple multiregional probabilistic population projections. Environment and Planning A 44(11): 2664–2686. doi:10.1068/a4533. 23236221 PMC3518036

[R82] Rees P and Turton I (1998). Investigation of the effects of input uncertainty on population forecasting. Paper presented at the GeoComputation 98 conference, Bristol, UK, September 17–19, 1998.

[R83] Rees P, Wohland P, Norman P, and Lomax N (2015). Sub-national projection methods for Scotland and Scottish areas: A review and recommendations Leeds: White Rose University Consortium, University of Leeds (Research report).

[R84] Reher DS (2004). The demographic transition revisited as a global process. Population, Space and Place 10(1): 19–41. doi:10.1002/psp.313.

[R85] Reher DS (2007). Towards long-term population decline: A discussion of relevant numbers. European Journal of Population 23(2): 189–207. doi:10.1007/s10680-007-9120-z.

[R86] Rele J (1987). Fertility levels and trends in India, 1951–1981. Population and Development Review 13(3): 513–530. doi:10.2307/1973137.

[R87] Rele J (1988). 70 years of fertility change in Korea: New estimates from 1916 to 1985. Asia-Pacific Population Journal 3(2): 29–54. 12281654

[R88] Rosero-Bixby L (2012). Estimates of fertility in the provinces of Costa Rica 1956–2011 [unpublished manuscript] San Jose: Centro Centroamerican de Poblacion, Universidad de Costa Rica.

[R89] Russian Federal State Statistics Service (2012). Age-specific fertility rates per regions for 1978–1979, 1982–1983, 1984–1985, 1986–1987, 1988–1989 Moscow: Russian Federal State Statistics Service.

[R90] Ševčíková H, Alkema L, and Raftery AE (2011). bayesTFR: An R package for probabilistic projections of the total fertility rate. Journal of Statistical Software 43(1): 1–29. doi:10.18637/jss.v043.i01. PMC5096741 27818617

[R91] Shorter F, Macura M, and the Panel on Turkey (1982). Trends in fertility and mortality in Turkey, 1935–1975 Washington, D.C.: National Academy Press.

[R92] Smith SK and Sincich T (1988). Stability over time in the distribution of population forecast errors. Demography 25(3): 461–474. 3234579

[R93] Smith SK and Tayman J (2004). Confidence intervals for population forecasts: A case study of time series models for states. Paper presented at the Population Association of America Meeting, Boston, USA, April 1–3, 2004.

[R94] State Statistics Service of Ukraine (2012). Population. [electronic resource] Kiev: State Statistics Service of Ukraine http://database.ukrcensus.gov.ua/MULT/Database/Population/databasetreeen.asp and http://www.ukrstat.gov.ua/druk/katalog/nasel/nasel2010.zip.

[R95] Statistical Centre of Iran (2001). Estimation of levels and patterns of fertility in Iran: With application of own-children method (1972–1996) Tehran: Statistical Centre of Iran.

[R96] Statistical Office of the Republic of Serbia (2012). Demographic yearbook 2002 and 2003: Table 2–11: General, specific and total fertility rates, 1950–2003, by regions Belgrade: Statistical Office of the Republic of Serbia, Division of Demography.

[R97] Statistical Office of the Republic of Slovenia (2012). Basic data on total fertility rates, statistical regions, Slovenia, annually for 2002 onward. [electronic resource] Ljubljana: Statistical Office of the Republic of Slovenia http://pxweb.stat.si/pxweb/Dialog/varval.asp?ma=05J2008Eti=path=../Database/Demographics/05population/30Fertility/1005J20FertilityREOBC/lang=1.

[R98] Statistical Office of the Slovak Republic (2012). Age-specific live births, female population, and fertility rates for 1996–2010 by regions (NUTS 3) and the Slovak Republic (Kópia - up hmr cmr 1996–2010 kraje sj.xls) Bratislava: Statistical Office of the Slovak Republic.

[R99] Statistics Austria (2012). Geborene: Langfristiger Trend. [electronic resource] Vienna: Statistics Austria http://statistik.at/webde/statistiken/menschenundgesellschaft/bevoelkerung/geborene/index.html.

[R100] Statistics Belgium (2012). Tableaux sur les naissances et fécondité: Table 24: Taux de fécondité selon l’âge des femmes atteint dans l’année, de 15 á 49 ans pour le pays et les regions (NI 03.24 historique FR v4.xls). [electronic resource] Brussels: Statistics Belgium, Direction générale Statistique et Information économique: Direction thématique Société http://statbel.fgov.be/fr.

[R101] Statistics Canada (2012). Births and total fertility rate, by province and territory. [electronic resource] Ottawa: Statistics Canada https://www.statcan.gc.ca/tables-tableaux/sum-som/l01/cst01/hlth85b-eng.htm.

[R102] Statistics Canada (2014). Population projections for Canada (2013 to 2063), provinces and territories (2013 to 2038): Technical report on methodology and assumptions Ottawa: Statistics Canada (Statistics Canada Catalogue 91–620-X). http://www.statcan.gc.ca/pub/91-620-x/91-620-x2014001-eng.htm.

[R103] Statistics Denmark (2012). StatBank. [electronic resource] Copenhagen: Statistics Denmark http://www.statbank.dk/statbank5a/default.asp?w=1280.

[R104] Statistics Estonia (2012). Main demographic indicators. [electronic resource] Tallinn: Statistics Estonia http://pub.stat.ee/px-web.2001/IDatabas/Population/01Populationindicatorsandcomposition/02Maindemographicindicators/02Maindemographicindicators.asp.

[R105] Statistics Finland (2012). Total fertility rate by region 1987–2011. [electronic resource] Helsinki: Statistics Finland http://tilastokeskus.fi/indexen.html.

[R106] Statistics for Wales (2017). Sub-national projections for Wales Cardiff: Welsh Government (Technical report). http://gov.wales/docs/statistics/2017/171019-local-authority-population-projections-technical-en.pdf.

[R107] Statistics Korea (2012). KOSIS 1970–2011 statistics table for live births, crude birth rate and total fertility rate by province. [electronic resource] Seoul: Statistics Korea http://kosis.kr/statHtml/statHtml.do?orgId=101tblId=DT1B81A21.

[R108] Statistics Norway (2012). StatBank Table 08556: Total fertility rate and age-specific fertility rates for 5-year periods. [electronic resource] Oslo: Statistics Norway http://www.ssb.no/en/table/08556.

[R109] Statistics of Japan (2012). 2010 vital statistics of Japan: Volume 1: Natality, table 4.5: Trends in total fertility rates by each prefecture for 1960–2010. [electronic resource] Tokyo: Ministry of Health, Labour and Welfare http://www.e-stat.go.jp/SG1/estat/CsvdlE.do?sinfid=000012659212.

[R110] Statistics Portugal (2012). Demographic indicators: Total fertility rate (no.) by place of residence (NUTS – 2002). [electronic resource] Lisboa: Statistics Portugal https://ine.pt/xportal/xmain?xpid=INExpgid=ineindicadoresindOcorrCod=0000600contexto=ptiselTab=tab10.

[R111] Statistics Sweden (1999). Befolkningsutvecklingen under 250 år: Historisk statistik för Sverige [Population development in Sweden in a 250-year perspective] Stockholm: Statistiska centralbyrån.

[R112] Statistics Sweden (2012a). Population by region, marital status, age and sex for 1968–2011. [electronic resource] Stockholm: Statistiska centralbyrån http://www.statistikdatabasen.scb.se/pxweb/en/ssd/STARTBEBE0101BE0101A/BefolkningNy/?rxid=86abd797-7854-4564-9150-c9b06ae3ab07.

[R113] Statistics Sweden (2012b). Live births by region, mothers age and childs sex for 1968–2011. [electronic resource] Stockholm: Statistiska centralbyrån http://www.statistikdatabasen.scb.se/pxweb/en/ssd/STARTBEBE0101BE0101H/FoddaK/?rxid=86abd797-7854-4564-9150-c9b06ae3ab07.

[R114] Stoto MA (1983). The accuracy of population projections. Journal of the American Statistical Association 78(381): 13–20. doi:10.1080/01621459.1983.10477916. 12265583

[R115] Tayman J (2011). Assessing uncertainty in small area forecasts: State of the practice and implementation strategy. Population Research and Policy Review 30(5): 781–800. doi:10.1007/s11113-011-9210-9.

[R116] Tayman J, Schafer E, and Carter L (1998). The role of population size in the determination and prediction of population forecast errors: An evaluation using confidence intervals for subcounty areas. Population Research and Policy Review 17(1): 1–20. 10.1023/A:1005766424443.

[R117] Tomlinson R (1985). The ‘disappearance’ of France, 1896–1940: French politics and the birth rate. Historical Journal 28(2): 405–415. doi:10.1017/S0018246X00003198. 11617076

[R118] UNFPA (2011). Impact of demographic change in Thailand Bangkok: United Nations Population Fund.

[R119] United Nations (2013). World population prospects: The 2012 revision New York: United Nations.

[R120] United Nations (2015). World population prospects: The 2015 revision, probabilistic population projections New York: Population Division, Department of Economic and Social Affairs, United Nations.

[R121] United Nations (2017). World population prospects: The 2017 revision New York: Population Division, Department of Economic and Social Affairs, United Nations http://esa.un.org/unpd/wpp.

[R122] US Census Bureau (2016). Subnational projections toolkit: Methods: Projtfr32. [electronic resource] Suitland: US Census Bureau https://www2.census.gov/software/sptoolkit/documentation/projtfr32-methods.pdf.

[R123] US Census Office (1902). 1900 census: Volume III: Vital statistics, Part 1: Analysis and ratio tables. [electronic resource] Washington, D.C.: US Census Office https://www.census.gov/library/publications/1902/dec/vol-03-vital-stats.html.

[R124] Wanner P (2000). Caractéristiques des régimes démographiques des cantons suisses 1870–1996. In: Association Internationale des Démographes de Langue Française (ed.). Colloque international de la Rochelle, 22–26 septembre 1998 Paris: Presses Universitaires de France: 243–253.

[R125] Watkins SC (1990). From local to national communities: The transformation of demographic regimes in Western Europe, 1870–1960. Population and Development Review 16(2): 241–272. doi:10.2307/1971590.

[R126] Watkins SC (1991). From provinces into nations: Demographic integration in Western Europe, 1870–1960 Princeton: Princeton University Press.

[R127] Wilson C (2001). On the scale of global demographic convergence 1950–2000. Population and Development Review 27(1): 155–171. doi:10.1111/j.1728-4457.2001.00155.x. 18589488

[R128] Wilson C (2004). Fertility below replacement level. Science 304(5668): 207–209. doi:10.1126/science.304.5668.207c. 15073356

[R129] Wilson C (2011). Understanding global demographic convergence since 1950. Population and Development Review 37(2): 375–388. doi:10.1111/j.1728-4457.2011.00415.x. 18589488

[R130] Wilson C, Singh A, Singh A, and Pallikadavath S (2012). Convergence and continuity in Indian fertility: A long-run perspective, 1871–2008. Paper presented at the Annual Meeting of the Population Association of America, San Francisco, US, May 3–5, 2012 http://paa2012.princeton.edu/abstracts/120470.

[R131] Wilson T (2013). Quantifying the uncertainty of regional demographic forecasts. Applied Geography 42: 108–115. doi:10.1016/j.apgeog.2013.05.006.

[R132] Wilson T and Bell M (2007). Probabilistic regional population forecasts: The example of Queensland, Australia. Geographical Analysis 29(1): 1–25. doi:10.1111/j.1538-4632.2006.00693.x.

[R133] Yao X (1995). China fertility data collection Beijing: China Population Press.

